# Craniofacial studies in chicken embryos confirm the pathogenicity of human *FZD2* variants associated with Robinow syndrome

**DOI:** 10.1242/dmm.050584

**Published:** 2024-07-05

**Authors:** Shruti S. Tophkhane, Katherine Fu, Esther M. Verheyen, Joy M. Richman

**Affiliations:** ^1^ Life Sciences Institute and Faculty of Dentistry, University of British Columbia, Vancouver, BC V6T 1Z3, Canada; ^2^ Department of Molecular Biology and Biochemistry, Centre for Cell Biology, Development and Disease, Simon Fraser University, Burnaby, BC V5A 1S6, Canada

**Keywords:** WNT signaling, Intramembranous ossification, Chondrogenesis, Micromass cultures, Luciferase assays, RCAS virus

## Abstract

Robinow syndrome is a rare disease caused by variants of seven WNT pathway genes. Craniofacial features include widening of the nasal bridge and jaw hypoplasia. We used the chicken embryo to test whether two missense human *FZD2* variants (*1301G>T*, p.Gly434Val; *425C>T*, p.Pro142Lys) were sufficient to change frontonasal mass development. *In vivo*, the overexpression of retroviruses with wild-type or variant human *FZD2* inhibited upper beak ossification. In primary cultures, wild-type and variant human *FZD2* significantly inhibited chondrogenesis, with the *425C>T* variant significantly decreasing activity of a SOX9 luciferase reporter compared to that for the wild type or *1301G>T*. Both variants also increased nuclear shuttling of β-catenin (CTNNB1) and increased the expression of TWIST1, which are inhibitory to chondrogenesis. In canonical WNT luciferase assays using frontonasal mass cells, the variants had dominant-negative effects on wild-type *FZD2*. In non-canonical assays, the *425C>T* variant failed to activate the reporter above control levels and was unresponsive to exogenous WNT5A. This is the first single amino acid change to selectively alter ligand binding in a FZD receptor. Therefore, *FZD2* missense variants are pathogenic and could lead to the altered craniofacial morphogenesis seen in Robinow syndrome.

## Teaser

**Summary:** Gain-of-function studies on human *FZD2* missense variants associated with Robinow syndrome lead to increased facial width, altered WNT signaling and inhibition of beak skeletogenesis in chicken embryos.

**Research Simplified**

Robinow syndrome is a rare disorder that affects the development of the skeleton, resulting in characteristic short stature and abnormalities in head and facial (craniofacial) bones. Variants in genes that regulate the Wnt signalling pathway, which is important for correct bone development, are known to cause Robinow syndrome. Of these, Frizzled2 (*FZD2*), coding for a receptor that transduces Wnt signals into cells to regulate their growth, is the second most common Robinow syndrome-causing gene. However, a detailed understanding of the impact of *FZD2* variants on the development of Robinow syndrome is missing.

In this study, the authors used chick embryos to investigate the impact of two *FZD2* variants on craniofacial development. Here, they found that expression of either *FZD2* variant resulted in the development of craniofacial abnormalities akin to those seen in Robinow syndrome, such as reduced bone formation and increased width of the nose and nasal cavities. Furthermore, the authors demonstrated that variant *FZD2* also inhibits the growth and formation of cartilage, and that this is due to altered activation of the Wnt signalling pathway.

Thus, this study successfully mimicked the craniofacial abnormalities caused by *FZD2* variants in Robinow syndrome. By enhancing our understanding of the underlying mechanisms of this rare disease, this work will be informative for future studies aimed at developing therapeutic interventions for Robinow syndrome.

## INTRODUCTION

Multiple syndromes include craniofacial dysmorphology as part of the clinical presentation. Studying human genetic diseases sheds light on gene function in morphogenesis, patterning and differentiation. In this study, we focused on Robinow syndrome, a rare skeletal dysplasia syndrome (1:500,000 live births) that primarily affects the skeleton of the face and limbs (Mazzeu and Brunner, 2020). Robinow syndrome was first reported in 1969 in a family with dwarfism and wide-spaced eyes or hypertelorism (Robinow et al., 1969). Approximately 250 cases of Robinow syndrome have been reported so far in the literature (Schwartz et al., 2021; Suresh, 2008). Interestingly, the seven genes associated with the pathogenesis of Robinow syndrome lie in the Wingless-related Integration site-1 (WNT) pathway (Table S1). Of these, receptor tyrosine kinase-like orphan receptor 2 (*ROR2*) and nucleoredoxin (*NXN*) are linked to autosomal recessive Robinow syndrome (White et al., 2018; Zhang et al., 2022), whereas dishevelled genes (*DVL1*, *DVL2* and *DVL3*), *WNT5A* and Frizzled2 (*FZD2*) have autosomal dominant inheritance (autosomal dominant Robinow syndrome) (Table S1) (Nagasaki et al., 2018; Person et al., 2010; Saal et al., 2015; White et al., 2015, 2018, 2016; Zhang et al., 2022). The primary phenotypes of Robinow syndrome include facial anomalies (hypertelorism, broad forehead, flat nasal bridge) (Beiraghi et al., 2011; Conlon et al., 2021; Kaissi et al., 2020; Sakamoto et al., 2021), limb shortening (Abu-Ghname et al., 2021; Zhang et al., 2022) and genital anomalies (males, small and buried penis; females, hypoplastic labia majora, small clitoris) (Patton and Afzal, 2002; Roifman et al., 2019). Despite high genetic heterogeneity, individuals with Robinow syndrome have similar clinical presentations (Mazzeu et al., 2007), which suggests that, ultimately, the genes share a common, indirect, downstream mediator in the WNT signaling pathway. Previously, our laboratory has investigated the effects of autosomal dominant Robinow syndrome *WNT5A* variants on jaw (Hosseini-Farahabadi et al., 2017) and limb (Gignac et al., 2019) development, and the effects of *DVL1* variants on limb development (Gignac et al., 2023). Here, we focused on two autosomal dominant Robinow syndrome *FZD2* variants and how they affect craniofacial development and WNT signaling.

WNTs are secreted glycoproteins that trigger the (1) canonical or β-catenin (CTNNB1)-dependent (Nusse and Clevers, 2017; Rim et al., 2022) and (2) several non-canonical or β-catenin-independent pathways (Angers and Moon, 2009; Lojk and Marc, 2021; Riquelme et al., 2023; Rogers and Scholpp, 2022). Most of the genes implicated in the pathogenesis of Robinow syndrome function in the non-canonical c-Jun N-terminal kinase (JNK)/planar cell polarity (PCP) pathway except for *FZD2*, *DVL1*, *DVL2* and *DVL3*, which also operate in the canonical/β-catenin-mediated WNT pathway. In the canonical pathway, WNT ligands bind to FZD and low-density lipoprotein receptor-related protein 5/6 (LRP5/6) receptors, which results in the recruitment of DVLs and destabilization of the β-catenin destruction complex. The free β-catenin accumulates in the cytoplasm and is subsequently translocated into the nucleus. In the nucleus, β-catenin forms a complex with the T-cell factor/lymphoid enhancer factor (TCF/LEF) family of transcription factors and activates transcription of WNT target genes (Nusse and Clevers, 2017). The translocation of β-catenin to the nucleus is a crucial signaling step in the WNT pathway and is often used as a readout of the active canonical WNT pathway (Anthony et al., 2020; Valenta et al., 2012). In the non-canonical JNK/PCP pathway, the WNT ligands bind with either FZD-ROR heterodimers (Green et al., 2014) or ROR homodimers, leading to the recruitment of DVLs, which act as a branch point for two small GTPase (RAC and RHO) pathways (van Amerongen et al., 2012). The RAC-mediated pathway involves the activation of JNK, which directly regulates cell polarity (Habas et al., 2003; Topczewski et al., 2011; Widelitz, 2005). As multiple genes are involved, the pathogenesis of Robinow syndrome could result from an imbalance of either branch of WNT signaling pathways, as we recently reported for *DVL1* variants (Gignac et al., 2023) and others for reported variants in *FZD2* (Liegel et al., 2023; Zhu et al., 2023).

FZD2 belongs to the FZD family of ten transmembrane receptors, which exhibit redundant functions (Schulte, 2010). Our laboratory and others have shown that *FZD2* is expressed abundantly in the developing face (Geetha-Loganathan et al., 2009; Yu et al., 2010, 2012). The extracellular regions of all FZDs consist of an N-terminal signal sequence followed by a highly conserved cysteine-rich ligand-binding domain (CRD). The CRD is linked to the seven-pass transmembrane domain consisting of three extracellular and three-intracellular loops and a C-terminus. The third intracellular loop and C-terminus are essential for interaction with DVLs (Fig. S1) (Schulte, 2010). *FZD2* is crucial for embryogenesis, cell polarity, cell proliferation and many other processes in developing and adult organisms (Huang and Klein, 2004; Wang et al., 2016). *FZD1*, *FZD2* and *FZD7* share 75% sequence homology and have substantial redundancy (Yu et al., 2010, 2012). *Fzd2^−/−^* null mice have fully penetrant cleft palate and craniofacial, cardiac and neural tube defects (Yu et al., 2010) The phenotypes were more severe in double knockout mice (*Fzd1^−/−^;Fzd2^−/−^*, *Fzd2^−/−^;Fzd7^−/−^*). Two frameshift mutations in the DVL-binding domain of *Fzd2* were created. A single-nucleotide insertion (extra guanine between c.1656 and c.1657) resulting in a frameshift in the DVL interaction domain (KTxxxW) in *Fzd2* (*Fzd2^INS/INS^*) caused fully penetrant cleft palate in mice (Zhu et al., 2023). Additionally, mice created to model the Robinow syndrome Fzd2^W548*^ variant (Fzd2^W553*^) are born with cleft palate along with other craniofacial deformities resulting in early perinatal lethality (Liegel et al., 2023). Genotype–phenotype correlation analysis revealed that individuals with *FZD2*-associated Robinow syndrome have milder craniofacial phenotypes than those with other gene variants (Zhang et al., 2021). Thus, the currently available animal models do not fully replicate the Robinow syndrome phenotypes.

*FZD2* is the second most common Robinow syndrome-causing gene (Zhang et al., 2022), and 17 patients with autosomal dominant Robinow syndrome with missense or truncating variants in *FZD2* have been reported (Table S2). Here, we investigate two missense *FZD2* variants using the chicken embryo model. The missense *FZD2* variants (c.*425C>T*, coding for p.Pro142Leu, and c.*1301G>T*, coding for p.Gly434Val) were chosen based on their location, unclear pathogenicity and facial phenotypes. The *425C>T* variant, termed as a variant of uncertain significance (VUS), lies in the CRD (Fig. S1A,B,B′). *425C>T* was identified in one compound heterozygous individual (*425C>T*, 1130G>A) with Robinow syndrome face and limb phenotypes (White et al., 2018). The father and two siblings of the proband also carried the *425C>T* variant and had short stature but no facial anomalies. The mother of the proband carried a truncating *FZD2* variant (c.*1130G>A*; p.Trp377*) and had a milder form of Robinow syndrome (short stature, broad forehead) (White et al., 2018). The second variant we characterized (*1301G >T*) was identified in the majority of patients with Robinow syndrome. *FZD2* variants that alter glycine 434 to either serine or valine have been reported in seven patients with Robinow syndrome (Nagasaki et al., 2018; Türkmen et al., 2017; Warren et al., 2018; White et al., 2018; Zhang et al., 2022). Glycine 434 present in the third intracellular loop of *FZD2* is highly conserved (Fig. S1A,C,D) and is essential for DVL binding (Tauriello et al., 2012). The elevated occurrence of pathogenic variants at glycine 434 can induce steric hindrance, leading to a reduced FZD–DVL affinity or stability (Punchihewa et al., 2009; Türkmen et al., 2017) (Fig. S1C,D). The *1301G>T* variant was associated with the pathogenesis of autosomal dominant omodysplasia type II (OMODII) (Nagasaki et al., 2018; Saal et al., 2015; Türkmen et al., 2017; Warren et al., 2018; Zhang et al., 2021). Given the significant overlap in phenotypes between OMODII and autosomal dominant Robinow syndrome, OMODII is currently recognized as part of autosomal dominant Robinow syndrome caused by *FZD2* variants (Zhang et al., 2022).

**Fig. 1.**
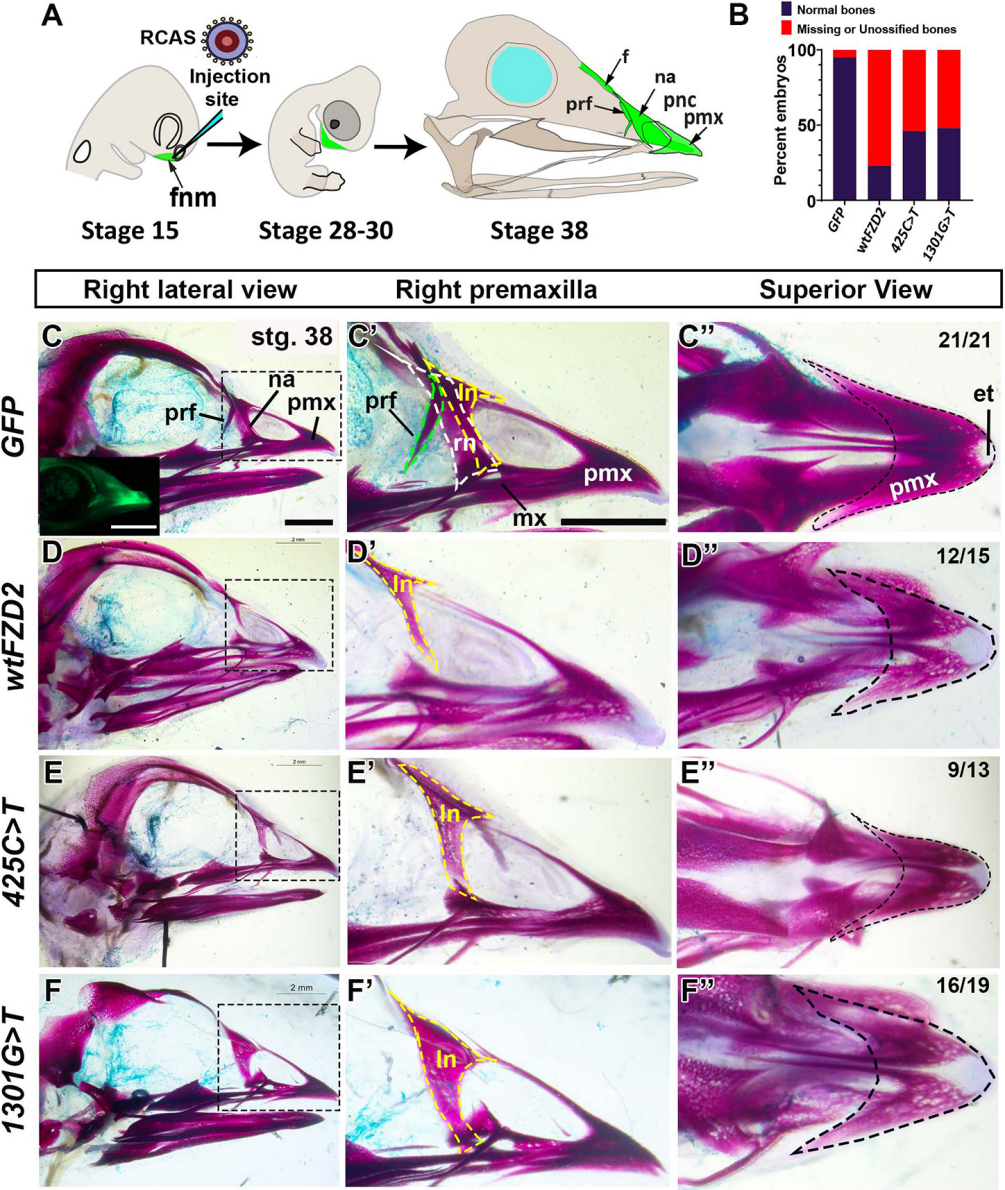
**Effects of *in vivo* overexpression of wild-type and variant h*FZD2*.** (A) RCAS viruses were microinjected into the right frontonasal mass at stage 15 (E2.5) as embryos are lying on their left side in the egg. Subsequent analysis of the upper beak was performed at stages 28 (E5.5) and 38 (E12.5). (B) The proportion of normal and abnormal embryos (missing/unossified bones) was significantly different between the h*FZD2* viruses and GFP controls as shown by Fisher's exact test. (C-F) Right side view of Alizarin Red staining (stage 38) of skulls that were injected with *GFP* (*n*=21), wt h*FZD2* (*n*=15) or h*FZD2* variants (*425C>T*, *n*=13; *1301G>T*, *n*=19). The inset in C shows the live GFP signal. Other viruses did not have a GFP tag and could not be detected in wholemount preparations. (C′-F′) Magnified right lateral views of the dotted regions in C-F to highlight the right prefrontal bone (green dashed line) and the right (white dashed line) and left (yellow dashed line) nasal bones. (D′-F′) All h*FZD2*-infected embryos lacked the right prefrontal and nasal bones. (C″-F″) View of the premaxilla (outlined with a dashed line). (D″-F″) The Alizarin Red stain was qualitatively fainter in h*FZD2* virus-infected embryos comped to *GFP-*infected controls. The light Alcian Blue stain was a technical error. et, egg tooth; f, frontal bone; fnm, frontonasal mass; ln, left nasal bone; mx, maxilla; na, nasal bone; pmx, premaxilla; pnc, prenasal cartilage; prf, prefrontal bone; rn, right nasal bone. Scale bars: 2 mm (C-F″).

We used the chicken embryo, a time- and cost-effective animal model, to study missense variants associated with dominant disease. Our approach involved introducing avian-specific replication-competent retroviruses [replication-competent avian sarcoma-leukosis virus (ASLV) long terminal repeat with a splice acceptor (RCAS)] (Hughes, 2004) containing wild-type (wt) or variant human (h) *FZD2* into the frontonasal mass (Fig. 1A), a facial prominence most affected in patients with Robinow syndrome. In avian species, the frontonasal mass gives rise to the prenasal cartilage, nasal septum, premaxillary bone, prefrontal bone and the egg tooth (Abramyan and Richman, 2018). In humans, the frontonasal mass contributes to midline structures of the face encompassing the bridge of the nose, the central region of the nose, the nasal septum, the philtrum and the central portion of the upper lip, including the premaxilla (Chen et al., 2013). Previous studies from our laboratory have demonstrated that autosomal dominant Robinow syndrome *WNT5A* (Gignac et al., 2019; Hosseini-Farahabadi et al., 2017) and *DVL1* variants (Gignac et al., 2023) have dominant-negative effects on chondrogenesis, leading to abnormal bone morphology. In this study, we uncover functional evidence that the *425C>T* VUS and the *1301G>T* variant retain activity and lead to inhibition of skeletogenesis. Therefore, both variants may be pathogenic in patients. We have also shown that the *425C>T* and *1301G>T* variants have surprisingly differing effects on WNT signal transduction in biochemical assays. These studies shed new light on ligand-binding specificity in the CRD of FZD receptors.

## RESULTS

Patients with autosomal dominant Robinow syndrome carry one normal and one variant copy of *FZD2.* Thus, our strategy was to overexpress the variant h*FZD2* in the targeted region, the frontonasal mass, alongside the endogenous chicken genome, to closely replicate the autosomal dominant genotype. In these experiments, we compared the effects of generalized increased levels of wt h*FZD2* to the two Robinow syndrome h*FZD2* variants (*425C>T* and *1301G>T*). The *GFP*-containing virus is a suitable control for overexpression studies as it does not affect development (Geetha-Loganathan et al., 2014; Hosseini-Farahabadi et al., 2017). Indeed, our previous work on autosomal dominant Robinow syndrome *DVL1* frameshift mutations (*1519ΔT*, *1529ΔG* and *1615ΔA*) (Gignac et al., 2023) and a *WNT5A* missense mutation (*248G>*C) (Gignac et al., 2019; Hosseini-Farahabadi et al., 2017) found that the variants retained activity and were sufficient to reduce size and change bone shape. The data was interpreted as follows: (1) if the missense h*FZD2* variants lacked function, then treated embryos should look like *GFP* controls; (2) if the variants retained activity, then they may give similar results to overexpression of h*FZD2*; (3) if the phenotypes were similar but more severe than those for wt h*FZD2*, then the variants cause a gain of function; and, lastly, (4) if the variant viruses induced *de novo* phenotypes compared to those for wt h*FZD2*, then this would suggest more complex functional alterations that would require additional analysis.

### Overexpression of wt or variant h*FZD2 in vivo* leads to abnormal patterning and inhibition of ossification

The viruses containing *GFP* (control), wt h*FZD2* or variants of h*FZD2* were overexpressed into the frontonasal mass at stage 15 [embryonic day (E) 2.5], before the cell fate is determined (Fig. 1A). The effects of exogenous genes on skeleton were studied at stage 38 (E12.5). By this stage, the upper beak bones derived from the frontonasal mass are fully ossified, allowing for comprehensive phenotypic analysis (Arnaout et al., 2021) (Fig. 1A). We were able to track the spread of virus in GFP-injected embryos to confirm targeting accuracy (Fig. 1C, inset). Embryos infected with *GFP*, wt h*FZD2* or h*FZD2* variants had normal beak outgrowth and shape (Table S3), and survival ranged between 64 and 78% (Table S4). *GFP*-infected skulls had all frontonasal mass derivatives (premaxilla, nasal and prefrontal bones) with robust Alizarin Red staining (Fig. 1C-C″; Fig. S2A-C). In contrast, we observed reduced Alizarin Red staining and agenesis of bones in the majority of embryos injected with wt h*FZD2* or h*FZD2* variants (Fig. 1B,D-F″, Fig. S2D-L). In particular, the prefrontal and nasal bones were missing, whereas the premaxillary bone showed reduced Alizarin Red staining compared to that in *GFP*-infected skulls (Fig. 1D-F″; Figs S2A-L and S3A-C, Table S3). The absence of some frontonasal mass derivatives and reduced ossification *in vivo* suggested that having generally higher levels of exogenous *FZD2*, whether wild-type or variant, inhibits intramembranous bone formation in chicken embryos. These data also showed that the h*FZD2* variants retained activity and are functional *in vivo*.

### Overexpression of variant h*FZD2* viruses causes an increase in frontonasal mass width mimicking Robinow syndrome

**Fig. 2.**
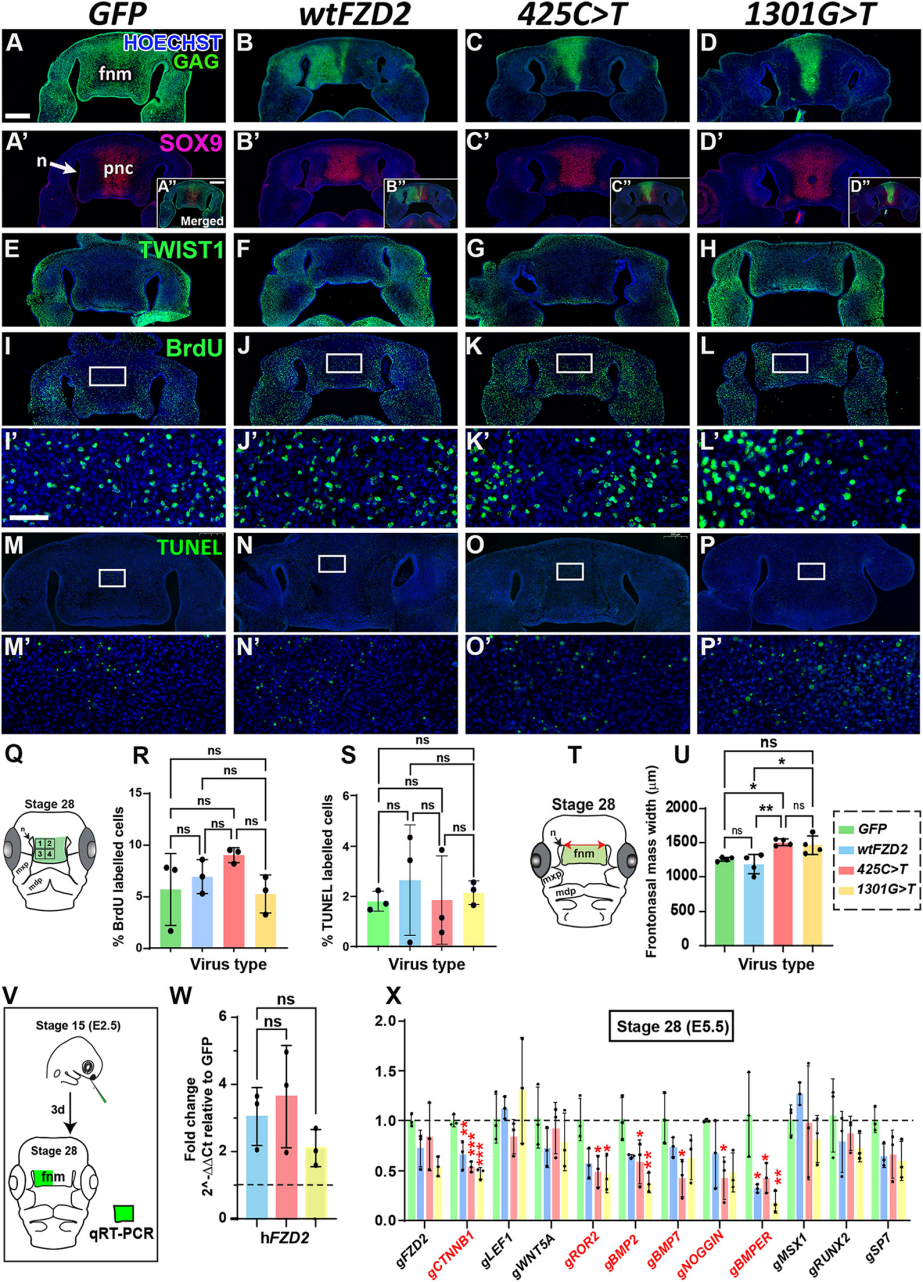
**Stage 28 embryos injected with *GFP*, wild-type h*FZD2* and h*FZD2* variants.** Frontal sections of heads were injected at stage 15 and fixed 72 h post injection at stage 28. (A-D) Sections stained for GAG show the viral spread (*n*=7). Sections with GAG staining shown in A-D are repeated in Fig. S4A,G,M,S. (Aʹ-Dʹ) The same sections co-labeled with anti-SOX9 antibody have staining in the prenasal cartilage (A″-D″) Insets for GAG and SOX9 double labeling. (E-H) Expression of TWIST1 in undifferentiated mesenchymal was similar in all conditions. The GAG staining for embryos in E-H can be found in Fig. S4B,G,M,T. (I-Lʹ) There were no significant differences in BrdU labeling between treatments. The GAG staining for embryos in I-L can be found in Fig. S4B,G,M,T. (M-Pʹ) TUNEL assay showed no difference in apoptosis between conditions. The GAG staining for embryos in M-P can be found in Fig. S4B,G,M,W. Please refer to Table S11 for a detailed list of embryos used for immunostaining and analysis at stage 28. Scale bars: 500 µm (A-L, insets); 200 µm (M-P); 50 µm (Iʹ-Pʹ). (Q) Schematic showing quadrants included in the BrdU- and TUNEL-positive cell analysis. (R,S) Quantification of the percentages of BrdU- and TUNEL-labeled cells shows that the h*FZD2* viruses did not affect proliferation and apoptosis. (T) Schematic showing measurement of frontonasal mass width taken between the superior aspect of the nasal slits. (U) The variant h*FZD2*-infected embryos were significantly wider compared to wt h*FZD2-* and *GFP*-infected embryos. (V) Schematic showing the right side of the frontonasal mass used for qRT-PCR analyses. Levels of expression were compared to *GFP* levels using the ΔΔCt method. (W) The average expression of h*FZD2* was 2- to 5-fold higher than that of *GFP*. (X) Effects of h*FZD2* viruses on the expression of WNT and BMP pathway genes. Genes with significantly different expression levels are shown in red. Error bars show mean±s.d. One-way ANOVA and Dunnett's test (compared to *GFP*) were used for comparisons. ns, not significant; *P<0.05; **P<0.01. fnm, frontonasal mass; mdp, mandibular prominence; mxp, maxillary prominence; n, nasal slit; pnc, prenasal cartilage.

Due to the size restraints of RCAS (2.5 kb) (Hughes, 2004; Loftus et al., 2001), a live tag (e.g. GFP) was not added to h*FZD2*. As a result, the h*FZD2* viral spread could not be verified. To link phenotypes to the presence of exogenous h*FZD2*, we collected embryos at stages 28 (E5.5) when significant morphological changes occur (Richman and Tickle, 1989, 1992; Wedden, 1987). This stage allowed verification of viral spread by immunostaining with an antibody against group-associated antigens (GAG) (Fig. 2A-D; Fig. S4A-X, Table S11) and quantification of viral gene expression changes by quantitative reverse transcription PCR (qRT-PCR) (Fig. 2X). Due to differences in the amount of virus injected between embryos, there was variability in viral spread. We selected embryos with strong GAG expression in the frontonasal mass for further characterization (Fig. S4A-X, Table S11).

Next, we tested the molecular effects of the h*FZD2* viruses. Serial sections selected from GAG-positive specimens were stained for the early chondrogenic marker SRY-box transcription factor 9 (SOX9) and TWIST-related protein 1 (TWIST1), a marker for undifferentiated mesenchymal cells. The wt and variant h*FZD2* did not affect the expression of SOX9 in the prenasal cartilage (Fig. 2Aʹ-Dʹ). The TWIST1-positive undifferentiated mesenchyme was also unaffected (Fig. 2E-H). In addition, cell proliferation (assessed by bromodeoxyuridine or BrdU labeling) (Fig. 2I-Lʹ,Q,R; Table S5) and apoptosis (assessed by terminal deoxynucleotidyl transferase dUTP nick end labeling or TUNEL) (Fig. 2M-Pʹ,S; Table S5) remained unchanged. In these early stages of development, we discovered that the h*FZD2* variants had *de novo* effects compared to wt h*FZD2* in controlling frontonasal mass morphogenesis.

We previously showed that frontonasal mass narrowing depends on Rho-associated kinase (ROCK) signaling, which is part of the non-canonical WNT signaling pathway (Danescu et al., 2021). At early stages of development, the frontonasal mass undergoes narrowing mesiodistally and elongates vertically (convergent extension) (Danescu et al., 2021; Diewert and Lozanoff, 1993). As individuals with Robinow syndrome have a wide-face phenotype, we hypothesized that the h*FZD2* variants interfere with the frontonasal mass-narrowing process. To test this, we measured the width of the frontonasal mass (distance between nasal slits) in the stage 28 embryos (schematic in Fig. 2T). Here, we found the first quantitative difference between the variants and wt h*FZD2*. Remarkably, embryos infected with h*FZD2* variants had on average a wider frontonasal mass compared to that in wt h*FZD2*- or *GFP*-infected embryos (Fig. 2U).

### Increased expression of wt or variant h*FZD2* has similar effects on RNA expression *in vivo*

Next, we quantified the effects of the viruses on gene expression *in vivo* (right half of the frontonasal mass) using qRT-PCR 72 h after injection (Fig. 2V). Notably, the addition of exogenous h*FZD2* (Fig. 2W) did not affect the expression of endogenous *Gallus gallus FZD2* (g*FZD2*) (Fig. 2X). As osteogenesis was inhibited by wt and variant h*FZD2*, we measured the RNA levels of WNT and bone morphogenic protein (BMP) pathway genes compared to their levels in *GFP* controls (Fig. 2X). In the presence of wt and variant h*FZD2*, *CTNNB1* (canonical pathway mediator) and *ROR2* (non-canonical WNT coreceptor) were significantly downregulated compared to their levels in *GFP* controls (Fig. 2X). In the BMP pathway, *BMPER* was downregulated by all h*FZD2* viruses. The *425C>T* variant downregulated *BMP2*, *BMP7* and *NOGGIN* (also known as *NOG*), and the *1301G>T* variant downregulated *BMP2* (Fig. 2X), compared to expression of these genes in *GFP* controls*.* Thus, RNA analysis further emphasized that overexpression of the wt h*FZD2* and variant h*FZD2* altered gene expression compared to that in *GFP* controls and that all the h*FZD2* viruses retained similar ability to alter gene expression, which correlates with similar effects of all h*FZD2* viruses on beak ossification.

### Variant h*FZD2* viruses inhibit chondrogenesis in frontonasal mass micromass cultures

The *in vivo* experiments showed that wt h*FZD2* or the h*FZD2* variants behaved similarly except that the variants inhibited facial narrowing. We used a complementary approach, i.e. primary facial mesenchyme placed into high-density culture to further test variants for differences compared to the wild-type gene. Micromass cultures are a well-established approach to study chondrogenesis in the face (Hosseini-Farahabadi et al., 2013; Richman and Crosby, 1990) and limbs (Weston et al., 2002). In addition, unlike *in vivo* where injections vary between each embryo, the level of virus infection and the culture environment can be standardized across experiments. The epithelium is excluded in micromass cultures, which makes it easier to study the effect of misexpression specifically on skeletogenic mesenchyme.

**Fig. 3.**
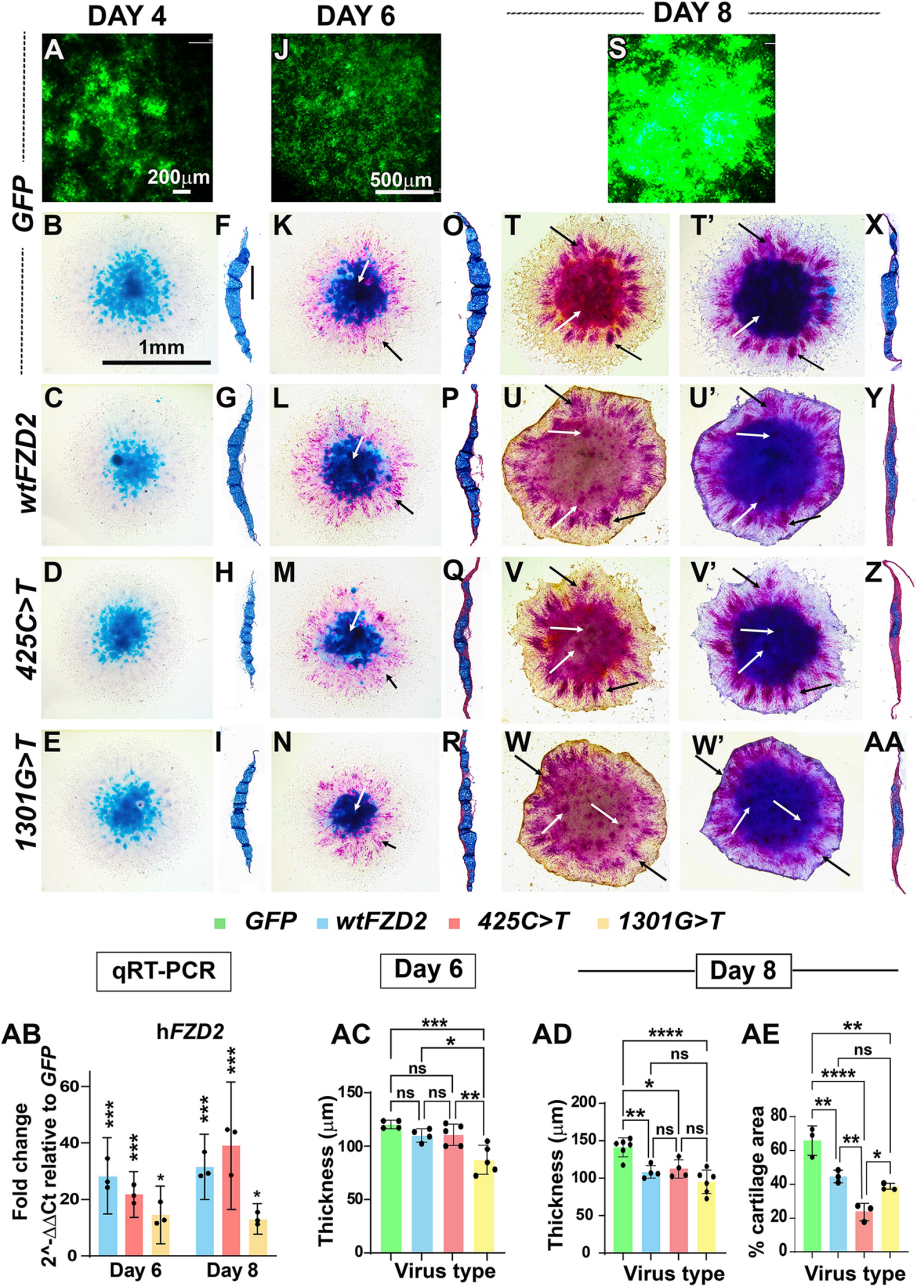
**Wholemount and histological analysis of day 4, 6 and 8 frontonasal mass micromass cultures.** (A,J,S) *GFP* virus spread in micromass cultures was observed on days 4 (A), 6 (J) and 8 (S). (B-E) Day 4 wholemount cultures stained with Alcian Blue showed normal cartilage patterns for the frontonasal mass. (F-I) Transverse sections (*n*=3) confirmed a cartilage sheet in the center of the cultures. (K-N) Wholemount cultures were stained with Alcian Blue (white arrows) and alkaline phosphatase to detect mineralization (black arrows). (O-R) Day 6 (*n*=7-9) histological sections stained with Alcian Blue and Picrosirius Red. (T-W) Before staining with Alcian Blue, cultures were photographed with just the alkaline phosphatase stain. *GFP*-infected cultures had alkaline phosphatase staining in the fringes (T, black arrows) and across the center (T, white arrows). In the h*FZD2*-infected cultures, there was strong alkaline phosphatase staining in the fringe mesenchyme (U-W, black arrows) and there was a lack of alkaline phosphatase in the center of the culture, suggesting that chondrocytes were not mineralized (U-W, white arrows). (T′-W′) After staining with Alcian Blue, the same cultures shown in T-W were rephotographed. Strong alkaline phosphatase staining was present in fringe mesenchyme (black arrows) and reduced intensity of Alcian Blue stain was visible (white arrows). (X-AA) h*FZD2* cultures were significantly thinner than *GFP* cultures. Scale bars: 200 μm (A); 1 mm (B; applies to C-E,K-N,T-W′); 100 μm (F; applies to G-I,O-R,X-AA); 500 μm (J; applies to S). (AB) qRT-PCR results showed similar levels of expression of the h*FZD2* genes (asterisks compare expression to *GFP* controls; *GFP* values are not shown). (AC) Measurements indicate that *1301G>T* day 6 cultures were already less thick compared to others. (AD) By day 8 (*n*=4-6), all h*FZD2* variant cultures failed to produce as much matrix as in the *GFP* cultures. (AE) All h*FZD2* cultures showed significantly reduced cartilage area over the sections. The *425C>T* virus inhibited cartilage matrix deposition more than wt h*FZD2* and *1301G>T*. Error bars show mean±s.d. One-way ANOVA with Dunnett's multiple comparison test was used. ns, not significant; *P≤0.05; **P≤0.01; ***P≤0.001; ****P≤0.0001.

In the micromass cultures, the viral spread could be observed as green fluorescence by staining with an anti-GAG antibody (Fig. 3A,J,S). Moreover, h*FZD2* transcript levels were comparable between the h*FZD2*-infected day 8 cultures (Fig. 3AB). The cultures were stained in wholemounts with Alcian Blue (to detect cartilage sheets) and alkaline phosphatase (to detect mineralization) (Fig. 3B-E,K-N,T-W′). On day 4, all viruses allowed the formation of Alcian Blue-stained cartilage sheets (Fig. 3B-E). This implied that wt h*FZD2* and h*FZD2* variants do not affect initial steps such as formation of cartilage condensations. At day 6, robust Alcian Blue-positive cartilage sheets were visible (Fig. 3K-N) and, for the first time, alkaline phosphatase-positive fibroblasts in the fringe of the culture were visible (Fig. 3K-N). There were no differences between the *GFP*- and h*FZD2*-infected cultures. The chondrogenesis and mineralization had progressed in day 8 cultures. All the h*FZD2*-infected cultures had less red alkaline phosphatase staining in the center of the cultures compared to that in *GFP* controls (Fig. 3T-W), echoing the decreased ossification seen *in vivo* by all h*FZD2* viruses. There were no qualitative differences in the extent of Alcian Blue staining between *GFP* and h*FZD2* viruses (Fig. 3T′-W′). However, the differences in Alcian Blue or alkaline phosphatase staining could not be quantified from wholemount images; therefore, we turned to transverse sections of cultures in which the microscopic differentiation of chondrocytes could be assessed.

In day 4 cultures, there were no qualitative differences in the thickness of the culture or amount of cartilage (Fig. 3F-I; Table S7). By day 6, the *1301G>T* variant-infected cultures had failed to increase in thickness compared to that of the other cultures (Fig. 3O-R,AC). By day 8, wt and variant h*FZD2* cultures were similarly unable to increase in thickness compared to that of *GFP* cultures (Fig. 3X-AA,AD). We confirmed the lack of continued matrix synthesis as the proportion of area occupied by Alcian Blue was significantly lower in h*FZD2-*expressing cultures compared to that in *GFP-*expressing cultures (Fig. 3AE).

**Fig. 4.**
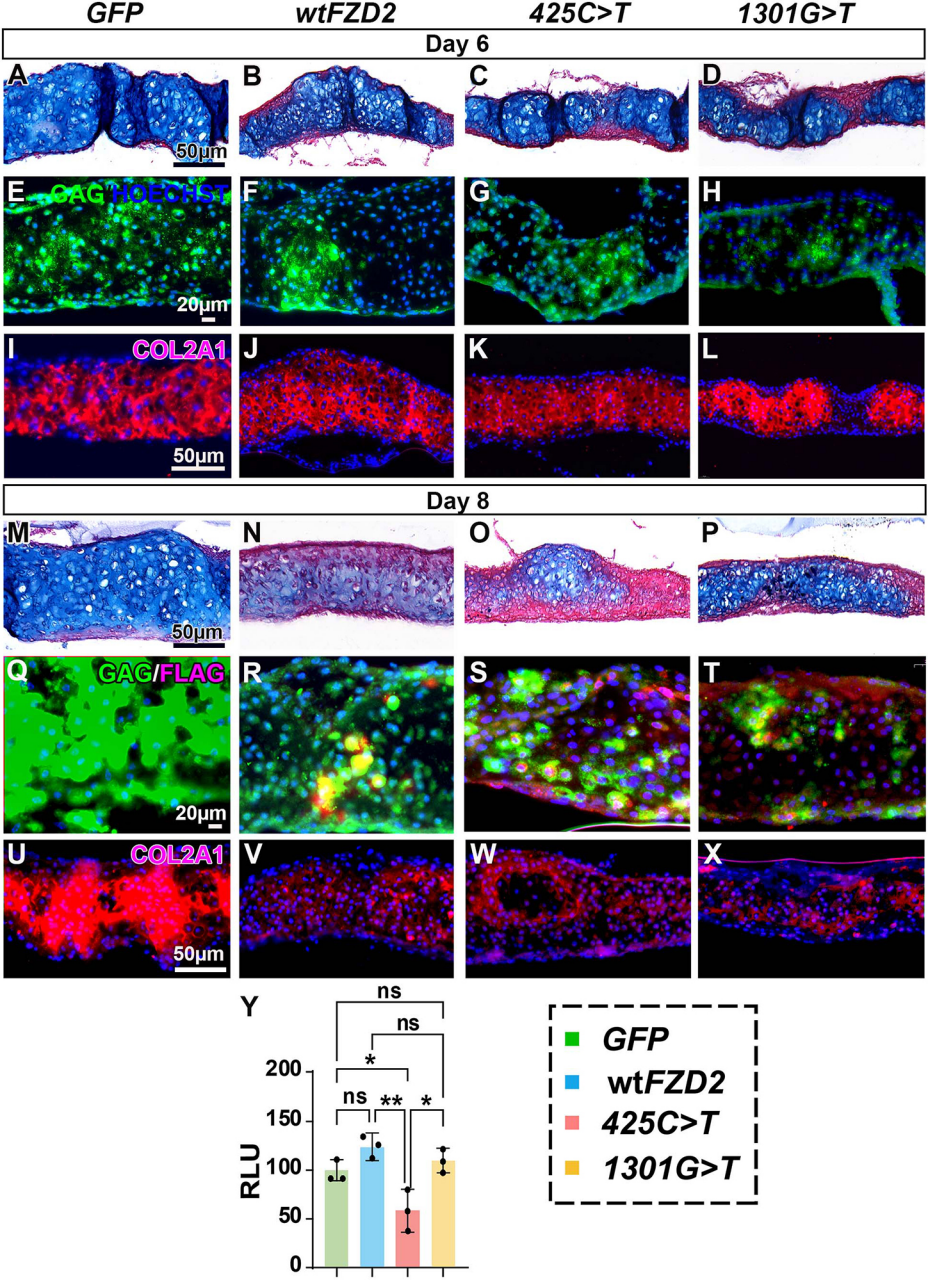
**Effects of h*FZD2* viruses on cartilage matrix *in vitro* on days 6 and 8.** (A-D) Day 6 cultures stained with Alcian Blue and Picrosirius Red. (E-H) Near-adjacent sections stained with anti-GAG antibody show viral spread (*n*=7-9). (I-L) Near-adjacent sections have comparable expression of cartilage matrix protein (type II collagen; COL2A1). (M-P) Day 8 cultures (*n*=4-6) infected with h*FZD2* viruses have reduced Alcian Blue staining and thickness compared to *GFP*-infected controls (see Fig. 3AD,AE). (Q-T) Viral spread shown with anti-GAG and anti-FLAG. hFZD2 proteins have a C-terminal FLAG tag. (U-X) All h*FZD2*-infected cultures show weak COL2A1 expression compared to that in *GFP*-infected controls. See Table S12 for a list of cultures analyzed. Scale bars: 50 µm (A,M,I,U; applies to B-D,J-L,N-P,V-X); 20 µm (E,Q; applies to F-H,R-T). (Y) Micromass cultures were transfected with SOX9 luciferase reporter 24 h after plating and read on day 3 just at the onset of chondrocyte specification. Cultures infected with *425C>T* had significantly reduced SOX9 reporter activity compared to that of cultures infected with other viruses. RLU, relative luminescence units (indicative of luciferase activity). Error bars show mean±s.d. One-way ANOVA and Tukey's post hoc test were used for multiple comparisons. ns, not significant; *P<0.05; **P<0.01.

We then turned to molecular readouts to determine whether there were differences in some of the proteins in the cartilage matrix. All day 6 and day 8 cultures showed comparable viral spread (Fig. 4E-H,Q-T). On day 6, there were areas of Alcian Blue staining in the centre of the culture (Fig. 4A-D) overlapping areas of type II collagen (COL2A1) expression (Fig. 4I-L). All areas examined had strong viral infection (Fig. 4E-H). There was no qualitative difference between type II collagen expression for wt h*FZD2*, h*FZD2* variants and *GFP* (Fig. 4I-L). On day 8, however, all h*FZD2*-infected cultures showed weak Alcian Blue staining (Fig. 4M-P) and COL2A1 staining compared to that in *GFP*-infected cultures (Fig. 4U-X). Thus, the data confirmed that the activity of the variants was similar to that of wt h*FZD2*, i.e. all failed to maintain the normal rate of matrix deposition.

### The *425C>T* variant fails to activate the SOX9 luciferase reporter

To further explore the mechanism for the reduction in cartilage, we used a reporter that measures the level of SOX9 transcriptional activity (Weston et al., 2002). *SOX9* has been shown to regulate COL2A1 in chondrocytes (Bell et al., 1997; Ng et al., 1997). We timed the transfection of the reporter so that the biochemical readout of luciferase activity was measured on day 3 of the culture. The cartilage condensations are first visible at 48 h post plating (Hosseini-Farahabadi et al., 2013) and 72 h is when transcriptional activity for chondrogenic genes is predicted to initiate. Interestingly, the *425C>T* virus failed to activate the SOX9 luciferase reporter compared to all other conditions (Fig. 4Y). This suggests that, for the *425C>T* variant, lower SOX9 expression could have reduced the number of chondrocytes that were specified, contributing to lower cartilage matrix deposition.

### h*FZD2* variants cause ectopic expression of nuclear β-catenin and TWIST1 *in vitro*

**Fig. 5.**
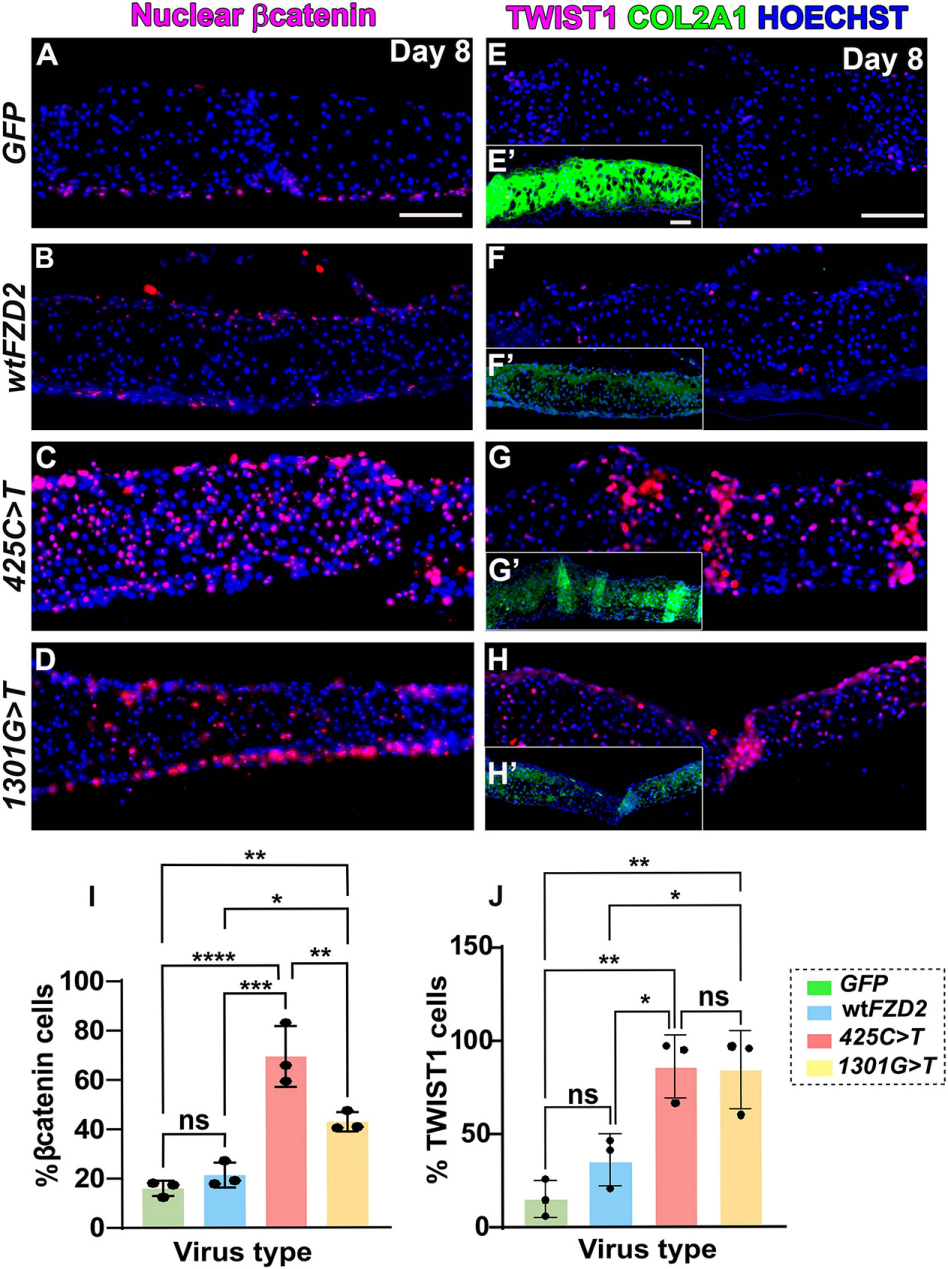
**h*FZD2* variants increased expression of nuclear β-catenin and TWIST1 on day 8.** (A,B) In *GFP-* or wt h*FZD2* virus-infected cultures, nuclear-β-catenin was found in fibroblasts at the superior and inferior edges. (C,D) In variant h*FZD2*-infected cultures, nuclear β-catenin was present in cartilage-forming regions. (E-Hʹ) Serial sections of the same cultures showing expression of TWIST1 and COL2A1 (E′-H′). Vertical brighter bands in G′,H,Hʹ are artifacts due to folds in the paraffin sections. See Table S12 for a list of cultures analyzed. Scale bars: 50 µm. (I) Quantification of β-catenin-positive cells showed that 40-60% of the cells in the variant h*FZD2*-infected cultures expressed nuclear β-catenin. (J) Quantification of TWIST1-positive cells showed an increase in the variant-infected cultures. Error bars show mean±s.d. One-way ANOVA and Tukey's post hoc test were used for multiple comparisons. ns, not significant; *P<0.05; **P<0.01; ***P<0.001; ****P≤0.0001.

To investigate the possible role of canonical WNT signaling in mediating the chondrogenic phenotypes of h*FZD2*-infected cultures, we examined the expression of nuclear β-catenin, a marker of active canonical WNT signaling. In *GFP-* and wt h*FZD2*-infected cultures, most nuclear β-catenin-expressing cells were found at the periphery of the cultures and excluded from the cartilage (Fig. 5A,B; Table S12). In contrast, in *425C>T-* and *1301G>T*-infected cultures, chondrocytes residing in the cartilage matrix also expressed nuclear β-catenin (Fig. 5C,D). Quantification of the proportion of cells expressing nuclear β-catenin revealed that *425C>T-* and *1301G>T*-infected cultures had a significantly higher number of nuclear β-catenin-positive chondrocytes compared to that in *GFP-* and wt h*FZD2-*infected cultures (Fig. 5I). This implies that the variants maintain canonical β-catenin mediated signaling longer than wt h*FZD2*. The levels of *CTNNB1* RNA were not significantly different between wt h*FZD2-* and variant h*FZD2-*infected day 8 cultures (Fig. S5B), but nuclear shuttling was affected as shown by antibody staining. The mechanism for decreased cartilage in wt h*FZD2*-infected cultures appears to be different than that in variant-infected cultures and does not involve the translocation of β-catenin to the nucleus.

As another readout of the β-catenin pathway, we examined whether chondrocytes expressed TWIST1, an established repressor of *SOX9* (Goodnough et al., 2012). Interestingly, we observed that the TWIST1-positive chondrocytes were more abundant in the *425C>T* and *1301G>T* variant-infected cultures compared to in *GFP*-infected cultures (Fig. 5E-H′,J). This suggests that the h*FZD2* variants cause β-catenin to enter the nucleus, which may activate the expression of the chondrogenic repressor TWIST1, ultimately inhibiting chondrogenesis *in vitro*.

We next analyzed whether h*FZD2* viruses reduced cell proliferation and increased cell death, thereby contributing to reduced thickness and chondrogenesis in the cultures. On day 6, BrdU labeled proliferating chondrocytes throughout the cartilage-forming area (Fig. S6A-D; *n*=7-9, Table S7). However, by day 8, there were almost no proliferating cells in the culture (Fig. S6E-H; *n*=4-6, Table S7), consistent with differentiation into chondrocytes. There was no visible difference in cell proliferation between the viruses. Apoptotic cells increased between day 6 and day 8 across all virus-infected culture conditions (Fig. S6I-P). Quantification revealed no wt or variant h*FZD2* effects on apoptosis at day 8 (Fig. S6Q,R). We also checked RNA expression of target genes in the WNT and BMP pathway but did not find significant differences between wt h*FZD2* and variant forms of h*FZD2* (Fig. S5A,B). In addition, there was no change in the expression of endogenous g*FZD2*, ruling out feedback loops (Fig. S5C).

Overall, the data indicate that the failure of cultures to grow in thickness is not related to the reduction of gene expression targets, proliferation or increased apoptosis but is likely due to a general inhibition of cartilage matrix synthesis. The ectopic expression of β-catenin and TWIST1 that is only seen in variant-infected cultures suggests that molecular inhibition of chondrogenesis also contributes to the reduced growth of the cultures.

### h*FZD2* variants have dominant-negative effects on wt h*FZD2* function

To clarify the activity levels of canonical and non-canonical WNT signaling, plasmids containing either wt or variant h*FZD2* were transiently transfected into frontonasal mass micromass cultures and luciferase assays were carried out. This approach ensured consistency in DNA levels and the signaling context of the facial mesenchyme was preserved. For a comparison, we also used HEK293T cells, a cell line commonly employed for luciferase assays.

**Fig. 6.**
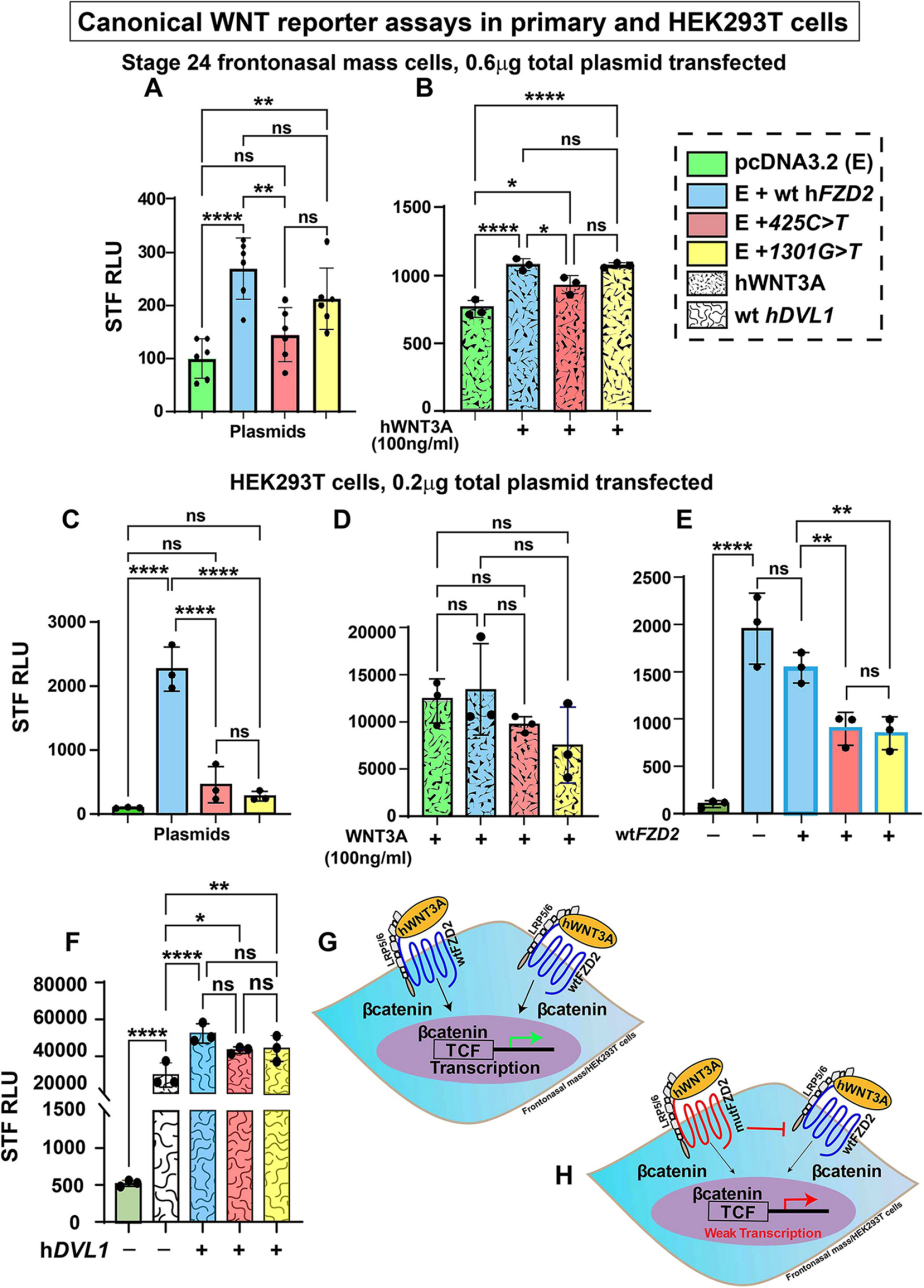
**Effects of h*FZD2* variants on Super TOPFlash luciferase activity in frontonasal mass cultures and HEK293T cells.** (A-F) β-catenin-mediated transcriptional activity was measured in stage 24 frontonasal mass micromass cultures (A,B) or HEK293T cells (C-F) using Super TOPFlash reporter (STF; 7× TCF/LEF-binding sites). (A,C) h*FZD2* variants showed significantly lower activation of STF compared to wt h*FZD2*. (B,D) Human (h) WNT3A (100 ng/ml) added 24 h post transfection increased STF activity for all cultures transfected with h*FZD2* plasmids. (E) In HEK293T cells, variants combined with equimolar amounts of wt h*FZD2* showed significantly less activation of STF compared to that for wt h*FZD2* alone. (F) When combined with wt h*DVL1*, a gene involved in signal transduction, all h*FZD2* plasmids showed equally increased STF activity that was significantly higher than that for h*DVL1* alone. These assays show that variants retain activity at similar levels to that for wt h*FZD2.* Three biological replicates and three technical replicates were performed, and the experiment was repeated three times. For A,B, 0.6 µg of plasmid (0.3 µg of pcDNA3.2+0.3 µg of *FZD2*) was used for transfection of frontonasal mass cultures. For C-F, 0.2 µg of plasmid (0.1 µg of pcDNA3.2/h*FZD2*+0.1 µg of h*DVL1*) was used for transfection of HEK293T cells. RLU, relative luminescence units (indicative of luciferase activity). Error bars show mean±s.d. One-way ANOVA and Tukey's post hoc test was used for multiple comparisons. ns, not significant; *P<0.05; **P<0.01; ***P<0.001; ****P≤0.0001. (G,H) Schematics showing the activity of wt *FZD2* (G) compared to that for *FZD2* variants (H). The dominant interference with the wt FZD2 receptor is shown by a red bar.

The Super TOPFlash reporter (STF) is a highly sensitive reporter that detects β-catenin/TCF-driven transcriptional activity (Veeman et al., 2003). In frontonasal mass cultures, the addition of wt h*FZD2* strongly activated STF compared to the empty vector (pcDNA3.2) (Fig. 6A). This suggested that h*FZD2* activated the canonical pathway by utilizing endogenous WNT ligands in chicken facial mesenchyme. STF activation was significantly lower for the *425C>T* variant compared to that for wt h*FZD2*; however, STF activation for the *1301G>T* variant was similar to that for wt h*FZD2* (Fig. 6A). The addition of human WNT3A (hWNT3A) strongly activated STF and STF levels were significantly elevated in the presence of all h*FZD2* plasmids compared to the empty vector, except that the *425C>T* variant showed significantly weaker activity compared to that for wt h*FZD2* (Fig. 6B).

In HEK293T cells, both h*FZD2* variants failed to activate STF in contrast to wt h*FZD2*, which strongly stimulated the reporter (Fig. 6C). When hWNT3A was added to HEK293T cells, there was similar activation of the pathway, regardless of which h*FZD2* plasmid was transfected (Fig. 6D).

FZD receptors form multimers in the cell membrane (Carron et al., 2003; Dann et al., 2001). Therefore, we wanted to test the effects of h*FZD2* variants on WNT signaling in the presence of the wt h*FZD2*. We used HEK293T cells for these assays as the both h*FZD2* variants had low STF activity compared to that for wt h*FZD2*. When an equimolar amount of either variant was transfected with wt h*FZD2*, we observed a significant reduction in the STF activity of wt h*FZD2* in the presence of h*FZD2* variants, suggestive of a dominant-negative effect (Fig. 6E, illustrated in Fig. 6G,H). These data mimic the heterozygous genotype (autosomal dominant) and suggest that in Robinow syndrome cases, the variant allele may dominantly inhibit the function of the normal allele.

The *1301G>T* variant lies in the DVL-binding domain and was proposed to affect the interaction between FZD2 and DVL. To test this, we measured the STF activity after transfection of HEK293T cells with h*FZD2* variants and wild-type human *DVL1* (h*DVL1*). On its own, h*DVL1* utilized the endogenous FZD receptors and significantly activated STF compared to the empty vector. STF activity increased significantly in the presence of wt or variants of h*FZD2* compared to wt h*DVL1* alone (Fig. 6F). There was no difference in STF activation between wt and variant h*FZD2* in the presence of *DVL1* (Fig. 6F)*.* Although the result with the extracellular variant *425C>T* was expected, the fact that the protein coded by the *1301G>T* variant did not interfere with WNT pathway activation in the presence of *DVL1* does not match the predictions made in human genetics studies (Türkmen et al., 2017; White et al., 2018) (Fig. S1). Further experiments (colocalization studies or protein–protein interaction studies by co-immunoprecipitation) are necessary to confirm the effects of the h*FZD2* variants on the FZD2–DVL interaction.

### The *425C>T* variant affecting the cysteine-rich domain causes a loss of function in the JNK/PCP pathway

**Fig. 7.**
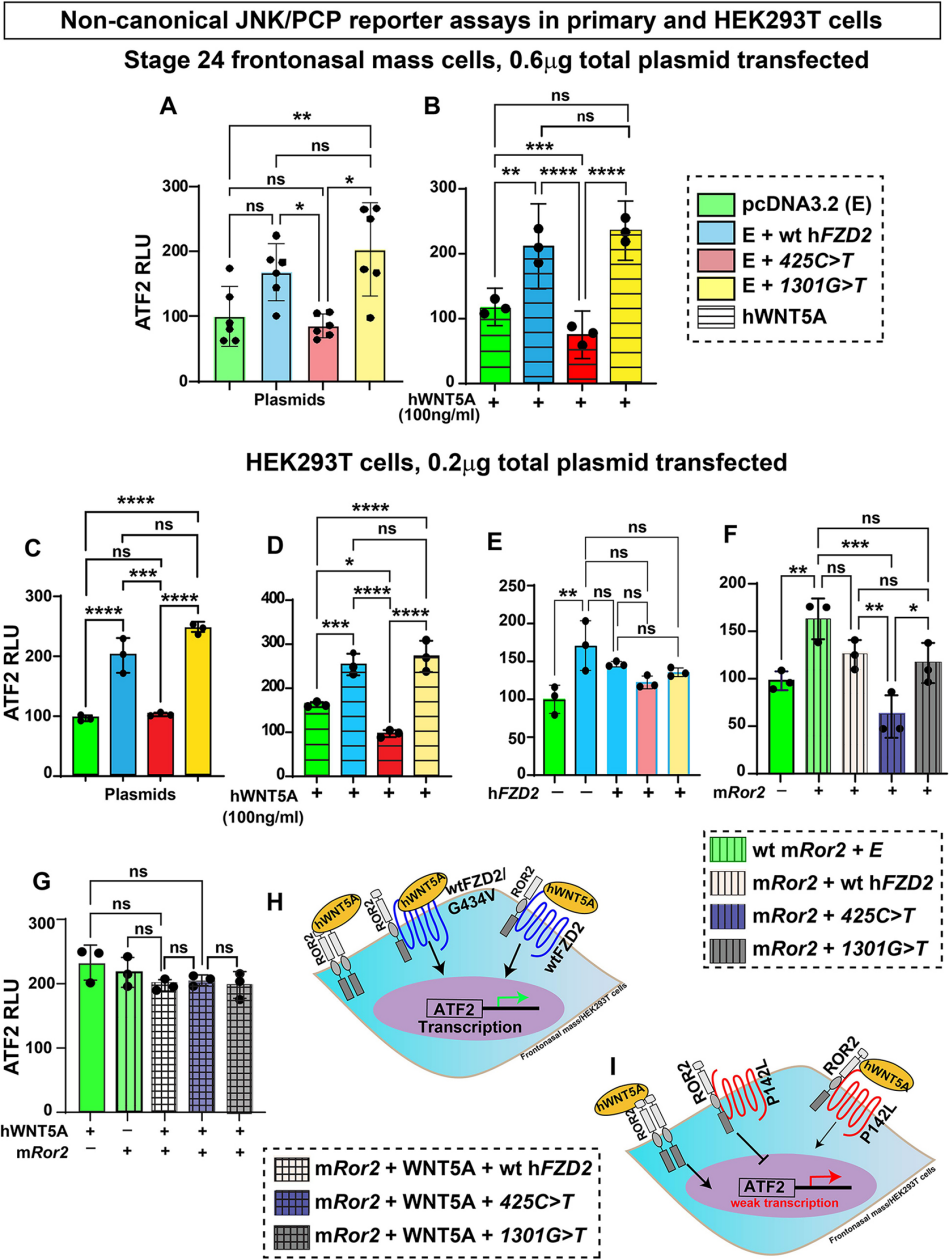
**Effects of h*FZD2* variants on the non-canonical WNT JNK/PCP pathway in frontonasal mass cultures and HEK293T cells.** (A-G) ATF2 luciferase was used to measure non-canonical WNT JNK/PCP pathway activity in stage 24 frontonasal mass micromass cultures (A,B) or HEK293T cells (C-G). (A,C) The *425C>T* variant showed significantly lower ATF2 activity compared to that for wt h*FZD2* and *1301G>T*. (B,D) Recombinant hWNT5A (100 ng/ml) was added 24 h post transfection. In the presence of wt h*FZD2* and the *1301G>T* variant, hWNT5A activated ATF2 more than that seen for control plasmids. However, hWNT5A was not able to activate the reporter in the presence of the *425C>T* variant. There was also significantly less activation by hWNT5A with *425C>T* compared to the empty plasmid. (E) All h*FZD2* variants combined in equimolar amounts with wt h*FZD2* (0.1 µg wt h*FZD2*+0.1 µg empty vector/wt h*FZD2*/variant h*FZD2*) showed comparable ATF2 activation. (F) When combined with m*Ror2*, the wt h*FZD2* and *1301G>T* plasmids showed similar ATF2 activation, but *425C>T* did not help to activate ATF2 by the mRor2 receptor. (G) WNT5A combined with m*Ror2* and wt h*FZD2* activated the reporter. As even the *425C>T* h*FZD2* variant was able to function in this assay, WNT5A is signaling going through Ror2. Three biological replicates and three technical replicates were performed, and the experiment was repeated three times. For A,B, 0.6 µg of plasmid (0.3 µg of pcDNA3.2+0.3 µg of plasmid *FZD2*) was used for transfection of frontonasal mass cultures. For C-F, 0.2 µg of plasmid (0.1 µg of pcDNA3.2/h*FZD2*+0.1 µg of m*Ror2*) was used for transfection of HEK293T cells. RLU, relative luminescence units (indicative of luciferase activity). Error bars show mean±s.d. Statistical analyses were performed with one-way ANOVA followed by Tukey's post hoc test multiple comparison test. ns, not significant; *P<0.05; **P<0.01; ***P<0.001; ****P≤0.0001. (H) Schematic showing JNK/PCP pathway activity of homodimers of ROR2, heterodimers of ROR2 and FZD2 (wt h*FZD2* or *1301G>T*). (I) Homodimers of ROR2 are able to function normally; however, heterodimers of ROR2 and the P142L (*425C>T*) variant of FZD2 are not able to signal.

As *FZD2* is known to participate in both canonical and non-canonical WNT pathways (Gordon and Nusse, 2006; Komiya and Habas, 2008), we used activating transcription factor 2 (ATF2) luciferase (Ohkawara and Niehrs, 2011) to measure JNK/PCP pathway activity. In the frontonasal mass micromass, the ATF2 reporter was activated (1.5-fold) by wt h*FZD2* compared to the empty vector (Fig. 7A). The *1301G>T* variant activated ATF2 to the same extent as wt h*FZD2*, whereas *425C>T* did not activate the reporter above basal levels (Fig. 7A). We next tested ATF2 activity in the presence of recombinant hWNT5A (100 ng/ml), a primarily non-canonical ligand. As expected, hWNT5A significantly activated ATF2 compared to the empty vector (Fig. 7B). When hWNT5A was combined with wt h*FZD2* or *1301G>T*, there were 2-fold increase in activity (Fig. 7B). It was striking that the *425C>T* variant failed to activate ATF2 in the presence of hWNT5A (Fig. 7B).

We replicated these results in HEK293T cells (Fig. 7C,D). Once again, we saw that the extracellular *425C>T* variant failed to utilize endogenous or exogenous WNT5A. We combined h*FZD2* variants with the wt h*FZD2* and found no evidence of synergistic or antagonistic effects between wt h*FZD2* and the variants in the JNK/PCP pathway, unlike the STF results (Figs 6E and 7E).

FZD2 also binds to ROR2 to form heterodimers that transduce signals from the WNT5A ligand (Cadigan and Waterman, 2012; Mikels et al., 2009; Nishita et al., 2010; Oishi et al., 2003). When mouse *Ror2* (m*Ror2*) was transfected into HEK293T cells, the ATF2 reporter was significantly activated compared to results for the empty vector (Fig. 7F). ATF2 reporter activity in the presence of m*Ror2* was equivalent to that for wt h*FZD2* and *1301G>T* (Fig. 7F,H). In contrast, the combination of *425C>T* and m*Ror2* failed to activate the ATF2 reporter (Fig. 7F,I). This suggests that mRor2 cannot form homodimers effectively in the presence of the *425C>T* variant. If homodimers could form, there would still be a strong ATF2 response.

To further investigate the formation of homodimers of mRor2, we added hWNT5A to the cells (Fig. 7G). The addition of hWNT5A appeared to overcome any block that was present when the *425C<T* variant was used. These data align with that of others where homodimers of mRor2 were shown to form in the presence of hWNT5A (Liu et al., 2008).

## DISCUSSION

Most genes that cause Robinow syndrome lie in the non-canonical WNT pathway except for DVLs and *FZD2*, which also function in the canonical WNT pathway. There are ten FZD receptors in humans, but interestingly, only h*FZD2* has been associated with the pathogenesis of Robinow syndrome (Lima et al., 2022; White et al., 2018). In this study, we characterized the effects of two missense h*FZD2* variants (*425C>T* and *1301G>T*) on face development and WNT signaling. We showed that the h*FZD2* variants inhibited embryonic facial morphogenesis, and these changes correlate with the wide-face phenotype, a characteristic of Robinow syndrome. Additionally, we also showed that both h*FZD2* variants have dominant-negative effects in the canonical pathway. Furthermore, we demonstrated that the *425C>T* variant (VUS) is pathogenic and fails to transduce signals in both branches of the WNT pathway.

### Effects of h*FZD2* variants on skeletogenesis *in vivo* and *in vitro*

Robinow syndrome primarily affects the skeleton of the face, limbs and vertebrae (Kaissi et al., 2020; Shayota et al., 2020). In this study, the wild-type and variants of h*FZD2* inhibited differentiation of bone in older embryos (E12.5) as well as mineralization in micromass cultures. We reported previously that overexpression of wt*WNT5A* or the Robinow syndrome *WNT5A^248G>C^* variant inhibited ossification in the mandible (Hosseini-Farahabadi et al., 2017). The effects of the h*FZD2* variants on bone mass or bone density in Robinow syndrome have not been characterized, so we cannot rule out the idea that there are clinically significant differences in bone quality.

The crosstalk between the WNT and BMP pathways is crucial for embryonic bone development and postnatal bone homeostasis (Itasaki and Hoppler, 2010; Lin and Hankenson, 2011). We showed a parallel downregulation of the WNT and BMP pathway genes caused by the h*FZD2* variants. This is the first study to establish a link between autosomal dominant Robinow syndrome h*FZD2* variants and the BMP pathway. Consistent with these data on reduced expression of BMP mediators, we found that the *425C>T* variant failed to activate the SOX9 luciferase reporter in day 3 micromass cultures. Further studies are needed to investigate the participation of the BMP pathway in mediating Robinow syndrome phenotypes.

In the facial mesenchyme, both the wild-type and variant forms of h*FZD2* produced similar effects, resulting in inhibition of cartilage matrix deposition. A detailed examination of the timeline in micromass culture and a comparison between the wt h*FZD2* and h*FZD2* variants revealed the following findings: (1) by day 2, both h*FZD2* variants exhibited limited activation of the canonical pathway; (2) on day 3, the *425C>T* variant failed to activate the SOX9 luciferase reporter; (3) between days 4 and 6, the h*FZD2* variants allowed cartilage condensations to form and secretion of cartilage matrix; and, lastly, (4) by day 8, quantifiable differences were observed between the variants and wild-type h*FZD2*. The most notable finding was the significant increase in the number of chondrocytes expressing nuclear β-catenin and TWIST1 surrounded by COL2A1-positive cartilage matrix. β-catenin, a key mediator of the canonical WNT pathway, has been shown to play a regulatory role in chondrogenesis (Akiyama et al., 2004; Day et al., 2005; Goodnough et al., 2012; Hosseini-Farahabadi et al., 2013). In the mouse cranial mesenchyme, β-catenin (*Ctnnb1*) binds to the promoter of *Twist1*. This interaction leads to the suppression of Sox9, suggesting a mechanism by which β-catenin inhibits chondrogenesis (Goodnough et al., 2012). Similarly, we show that in the chicken cranial mesenchyme, Robinow syndrome-associated h*FZD2* variants cause an ectopic increase in nuclear β-catenin and TWIST1 expression, alongside a decrease in cartilage matrix protein levels. This implies that the h*FZD2* variants utilize a different mechanism to disrupt chondrogenesis compared to wild-type h*FZD2*.

### Distinct effects of the h*FZD2* variants on WNT signaling pathways

**Fig. 8.**
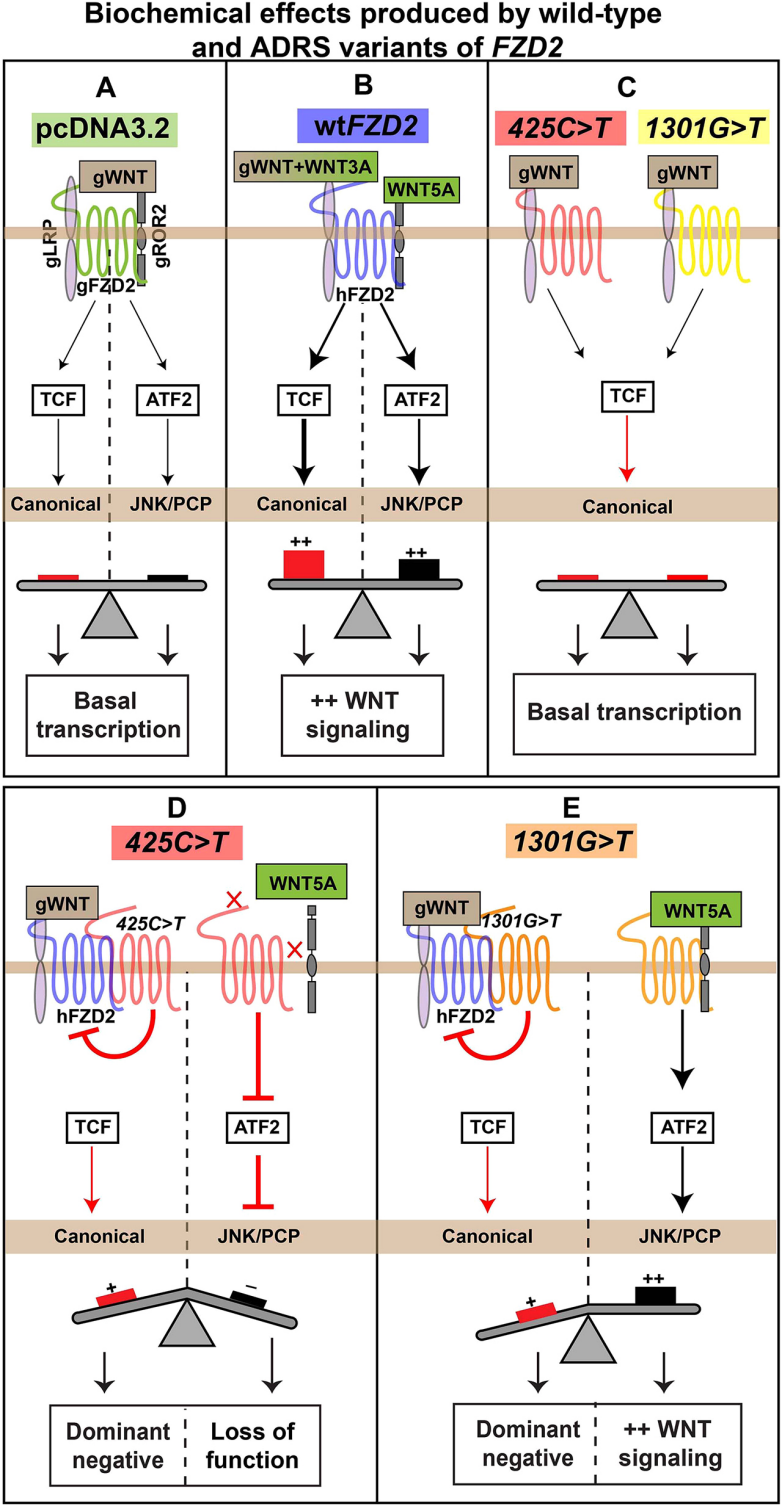
**Summary of signaling effects produced by wild-type h*FZD2* or h*FZD2* variants.** Schematic of biochemical activity of wild-type or variants of h*FZD2*. (A) Transfection of the empty vector (pcDNA3.2) in frontonasal mass mesenchyme or HEK293T cells showed basal level activity in both the canonical (Super TOPFlash reporter or STF, red) and JNK/PCP (ATF2 reporter, black) pathways. (B) Wild-type h*FZD2* (blue) significantly activated STF and ATF2. (C) The *425C>T* (red) and the *1301G>T* (yellow) variants weakly activated STF. (D) The *425C>T* variant, when combined with wild-type h*FZD2*, showed a dominant-negative effect on the activity of the wild type. When *425C>T* was combined with WNT5A or m*Ror2*, the JNK/PCP pathway was not activated. (E) The *1301G>T* variant also, when combined with wild-type h*FZD2*, showed a dominant-negative effect. The *1301G>T* variant by itself and combined with WNT5A and m*Ror2* activated the JNK/PCP pathway to the same extent as wild-type h*FZD2*. ADRS, autosomal dominant Robinow syndrome.

Our luciferase assays on the primary facial mesenchyme and HEK293T cells showed that both the h*FZD2* variants activated the canonical WNT pathway to the same extent as the empty vector (Fig. 8A,C) but were weaker compared to wt h*FZD2* (Fig. 8B,C). Both Robinow syndrome h*FZD2* variants have dominant-negative effects on the activity of wt h*FZD2* in the canonical β-catenin-mediated pathway (Fig. 8D,E). This finding is consistent with a study that found that *Fzd2^em1Rstot(D4)^* homozygotes (Fzd2^552fs*60^, frameshift in the Dvl interaction domain) decrease canonical WNT signaling as measured by a reduction in *Axin2* RNA levels (Liegel et al., 2023). Furthermore, *Fzd2^INS/INS^* mice also showed reduced expression of canonical *Axin2* (a canonical WNT target gene) in the limb buds (Zhu et al., 2023), possibly due to the failure of Dvl1 to interact with Fzd2. Additionally, the autosomal dominant Robinow syndrome rFzd2^p.TRP548*^-*GFP* variant was shown to reduce recruitment of human DVL2-FLAG in HEK293T cells (Saal et al., 2015).

The ATF2 reporter was specifically designed to read out JNK/PCP-dependent activation of the transcription factor ATF2 (Ohkawara and Niehrs, 2011). Although the ROR2-FZD7-WNT5A complex has been shown experimentally to activate the JNK-PCP pathway (Nishita et al., 2010), the ROR2-FZD2-WNT5A complex has not been studied. Here, we showed that when wt h*FZD2* was combined with either hWNT5A or m*Ror2*, there was no increase in ATF2 reporter activity compared to for h*FZD2* alone. Thus, FZD2 may not form dimers with mRor2. Indeed, other work showed that binding of WNT5A to ROR2 prompts homodimerization of ROR2, excluding FZD from the signalosome (Feike et al., 2010; Liu et al., 2007, 2008).

The variants each had different effects on the ATF2 reporter. Although the intracellular *1301G>T* variant activated ATF2 at similar levels as wt h*FZD2* in all assays, the extracellular *425C>T* variant failed to activate ATF2 in the presence of exogenous hWNT5A and/or m*Ror2*. The *425C>T* variant replaces proline 142 in the CRD with leucine. This proline is conserved across all ten FZD receptors. Here, we demonstrate that this proline 142 controls ligand specificity within the CRD. There have been very few structure–function studies involving point mutations in FZD receptors. Recently, molecular biosensors have been developed that could be used to measure the effect of single amino acid changes in FZDs on binding of WNT ligands (Kozielewicz et al., 2021). These studies are necessary to unlock the puzzle of WNT ligand binding (Dijksterhuis et al., 2015).

### The new role of *FZD2* in midface narrowing

Previous work from our laboratory showed that frontonasal mass narrowing in chicken embryos is dependent on small GTPase signaling mediated by ROCK (Danescu et al., 2021). In that study, we excluded oriented cell division or pressure from the expanding eyes as mechanisms for frontonasal mass narrowing. Indeed, the direct inhibition of ROCK with Y27632 prevented facial narrowing in the chicken embryo (stage 28, E5.5) (Danescu et al., 2021). Our present data extend these results and we find that two Robinow syndrome h*FZD2* variants also interfere with facial narrowing at the same stage of development. We showed that the *425C>T* variant is significantly weaker in activating the JNK/PCP pathway (JNK is downstream of RAC) (Qin et al., 2024). However, the *1301G>T* variant inhibited facial narrowing without affecting the ATF2 reporter. The reason could be that the *1301G>T* variant inhibits another branch of the non-canonical WNT pathway – the RHOA/ROCK-mediated pathway (Qin et al., 2024). In future experiments, it is necessary to evaluate the effects of the h*FZD2* variants specifically on the ROCK activity. All forms of Robinow syndrome have the same distinct facial features, hypertelorism and a broad nasal bridge (Zhang et al., 2022). Thus, our data suggest that lower PCP activity in the frontonasal mass at a specific time in development may be the common mechanism underpinning the phenotypes.

### Micromass cultures clarify variant-specific effects in signaling and differentiation

In our study, specific experiments did not show specific effects cased by the h*FZD2* variants compared to wt h*FZD2*, and these support a general gain of function due to the presence of h*FZD2*. This includes the *in vivo* reduced ossification phenotype and gene expression changes shown by the qRT-PCR, BrdU and TUNEL assays. We also have presented data that show specific effects of the variants *in vivo* (increased frontonasal mass width), *in vitro* micromass cultures (reduced thickness and amount of cartilage, increased TWIST1 and CTNNB1 expression) and luciferase assays (STF, ATF2, SOX9). Taken together, the most substantial evidence for changes in variant function are those performed on primary facial mesenchyme cells in micromass cultures and luciferase assays. Excluding the epithelium helped identify the effects of the variants on facial mesenchyme that could not be appreciated *in vivo*.

### Overexpression in chicken embryos complements mouse models for Robinow syndrome variants

The overexpression experiments in chicken embryos test whether a variant is sufficient to change the course of development. The results are complementary to mouse genetic models in which the variant is knocked into the equivalent location of the genome. In the mouse constitutive knock-in model, the spatiotemporal regulation of gene expression is conserved and mirrors the expression levels seen in the human autosomal dominant heterozygous cells. Indeed, the *Fzd2* knock-in model was recently published (Liegel et al., 2023). In this study, the Fzd2^W553*^ variant caused 100% lethality in the embryos, even in the heterozygous state. It was necessary to go to great lengths to produce F0 embryos, including electroporation of the oviduct. As animals could not be bred, there were limits to the number of mutant embryos available to study at different stages of development. Therefore, even though, in theory, the mouse knock-in model appears to be superior, unexpected problems can occur. There are other examples of lethality in heterozygous mice with constitutively expressed mutations, such as one with a missense mutation knocked into *Ifitm5*, which, in humans, causes type V osteogenesis imperfecta (Rauch et al., 2018). The mouse models may also have no phenotype unless bred to homozygosity. Therefore, with all these caveats for the mouse model, overexpression in the chicken embryo or other animal models (Gignac et al., 2023) is required to define the effects of the mutation fully. Furthermore, our chicken data suggest that other variants in h*FZD2* may be worth following up in the mouse based on their signaling defects and effects on cell differentiation.

Overall, this study provides a mechanistic understanding of the developmental, molecular, cellular and biochemical processes affected in patients carrying variants in *FZD2*. These discoveries broaden our understanding of the WNT pathway and will be helpful in future studies to develop therapeutic interventions for patients with Robinow syndrome.

## MATERIALS AND METHODS

### Chicken embryo model

White leghorn eggs (*Gallus gallus*) obtained from the University of Alberta, Edmonton, were incubated to the appropriate embryonic stages, based on the Hamilton–Hamburger staging guide (Hamburger and Hamilton, 1992). All experiments were performed on prehatching chicken embryos, which are exempt from ethical approval by the University of British Columbia Animal Care Committee and the Canadian Council on Animal Care.

### Cloning of h*FZD2* constructs

The open reading frame encoding the h*FZD2* sequence was purchased from GeneCopoeia (Rockville, MD, USA; clone #GC-S0193-B). Restriction-free cloning (Bond and Naus, 2012) was used to add the DNA sequence coding for a C-terminal FLAG tag (DYKDDDDK) into the wt h*FZD2* vector (pShuttle, GeneCopoeia) and a stop codon was added at the 3′ end of the tag. Two autosomal dominant Robinow syndrome-associated missense variants (*1301G>T* and *425C>T*) were knocked into the FLAG-tagged wt h*FZD2* (pShuttle) vector (Table S6). FLAG-tagged wild-type or variant h*FZD2* vectors (pShuttle) were then recombined into destination vectors [pcDNA 3.2/V5-DEST mammalian expression vector (Thermo Fisher Scientific, 12489019) and RCAS retroviral vector (gifted by Dr Stacie K. Loftus, National Human Genome Research Institute, National Institutes of Health, Bethesda, MD, USA; Loftus et al., 2001)] using Gateway LR clonase II enzyme mix (Thermo Fisher Scientific, #11791019) as described previously (Hosseini-Farahabadi et al., 2017). *GFP* was not fused to the 5′ end of h*FZD2* constructs due to the large size of *GFP* interfering with the localization of FZD2 to the cell membrane. The small size of the FLAG tag and its high hydrophilicity tend to decrease the possibility of interference with protein expression and function (Einhauer and Jungbauer, 2001). To create the RCAS viruses, a Gateway-compatible RCASBPY destination vector (gifted by Dr Stacie K. Loftus; Loftus et al., 2001) was used for recombination with the pShuttle vector using LR Clonase II (Loftus et al., 2001). It should be noted that the insert size for RCAS is restricted to 2.5 kb (Loftus et al., 2001). Due to the size restraints, a GFP tag (construct size of 730 bp) to track the viral spread could not be added.

### Growth of RCAS viral particles and viral titer

RCAS (Hughes, 2004; Loftus et al., 2001) plasmid DNAs (2.5 µg) encoding *GFP*, h*FZD2* or two h*FZD2* variants (*425C>T* and *1301G>T*) were transfected into the DF-1 immortalized chicken fibroblast cell line (American Type Culture Collection, #CRL-12203) using Lipofectamine 3000 (Thermo Fisher Scientific, L3000-008) following the manufacturer's guidelines. RCAS virus containing *GFP* insert (kindly provided by Stephen J. Gaunt, University of Cambridge, UK) served as a control in virus overexpression studies, as published (Geetha-Loganathan et al., 2014; Gignac et al., 2019, 2023; Hosseini-Farahabadi et al., 2013, 2017). DF-1 cells were cultured at 37°C and 5% CO_2_ in Dulbecco's modified Eagle medium (DMEM; Thermo Fisher Scientific, #1967497) supplemented with 10% fetal bovine serum (FBS; Sigma-Aldrich, #F1051) and 1% penicillin/streptomycin (Thermo Fisher Scientific, #15070-063). The cells were maintained in 100 mm culture dishes with medium changes every other day and passaged 1:2 two to three times per week using trypsin-EDTA (0.25%, Thermo Fisher Scientific, #25200-072). After 6 weeks of culturing, the viral particles were collected and centrifuged in a swing bucket SW28 rotor (Beckman Coulter, #97U 9661 ultracentrifuge) for 2.5 h (no brake) at 25,000 rpm (77,000 ***g***) at 4°C. The supernatant was carefully removed and the resulting pellet was resuspended in 50-100 µl Opti-MEM (Thermo Fisher Scientific, #319850962). This suspension was then incubated overnight at 4°C. The concentrated viral particles obtained were aliquoted (5 µl aliquots), rapidly frozen in methanol and dry ice, and stored at −80°C for future use (Goodnough et al., 2012).

To determine the viral titer, 50-60% confluent DF-1 fibroblasts were infected with serial dilutions of 2 μl of concentrated viral stock. After 36 h of virus incubation, cells were fixed in 4% paraformaldehyde (PFA) for 30 min. Immunocytochemistry with the anti-GAG antibody was performed on virus-treated cells (Table S9). The cells were permeabilized for 30 min with 0.1% Triton X-100, followed by blocking in 10% goat serum and 0.1% Triton X-100 and overnight incubation with the primary antibody (Table S9). Fluorescence images were captured using a Leica inverted microscope at 10× with a DFC7000 camera. The analysis of viral titer was done with the cell counter tool in ImageJ by determining the proportion of GAG-positive cells per milliliter in a 35 mm culture plate. Virus titer=number of GAG positive cells×(total area counted×number of cells expressing GAG)/2×1000 (Fig. S7).

### Chicken embryo injections

Fertilized eggs obtained from the University of Alberta were incubated in a humified incubator at 38°C until Hamilton–Hamburger stage 15 (E2.5) (Hamburger and Hamilton, 1951, 1992). Concentrated RCAS (titer ≥2×10^8^ IU/ml) retrovirus viral particles (∼5 µl) combined with Fast Green FCF stain (0.42%, Sigma-Aldrich, #F7252) (1 µl) were injected into the frontonasal mass (anatomic region bounded by the nasal slits) of stage 14-15 chicken embryos (25-28 somites) using glass filament needles (thin-wall borosilicate capillary glass with microfilament, A-M Systems, #615000) and a Picospritzer microinjector (General Valve Corp., #42311). The infection of embryos with RCAS at stage 15 (E2.5) was performed to ensure maximum infection of facial prominences (Geetha-Loganathan et al., 2009; Hosseini-Farahabadi et al., 2017). Due to accessibility, all injections were made into the right frontonasal mass as the chick embryos turn on their left side during development. The facial prominences form around stage 20, and the complex and temporally regulated patterning occurs between stages 20 and 29. The skeletal derivatives of the frontonasal mass are fully patterned and ossified between stages 34 and 40. The investigation encompassed multiple embryonic stages to comprehensively analyze these developmental processes. After overexpression of high-titer h*FZD2* viruses at stage 15, the retrospective determination of virus location was carried out on histological sections. These sections were stained with the anti-GAG antibody, which identifies specific proteins in the RCAS virus (Geetha-Loganathan et al., 2009; Gignac et al., 2023; Hosseini-Farahabadi et al., 2017), or an antibody against the FLAG sequence (Gignac et al., 2023; Tetenborg et al., 2020) (Table S9).

### Wholemount staining of skulls

To study skeletal elements, embryos were grown until stage 38 (10 days post injection, Table S3). The embryos were washed in 1× phosphate-buffered saline (PBS; 137 mM NaCl, 8.1 mM Na_2_HPO_4_, 2.7 mM KCl, 1.5 mM KH_2_PO_4_; pH 7.3) and fixed in 100% ethanol for 4 days. After removal of the eyes and skin, the embryos were transferred to 100% acetone for another 4 days. Subsequently, the heads were stained with a freshly prepared bone and cartilage stain (0.3% Alcian Blue 8GX, Sigma-Aldrich, #A5268) in 70% ethanol and 0.1% Alizarin Red S (Sigma-Aldrich, #A5533) in 95% ethanol, with one volume of 100% acetic acid and 17 volumes of 70% ethanol for 2 weeks on a shaker at room temperature. Following staining, the skulls were washed in water and cleared in a 2% KOH/20% glycerol solution on a shaker for 4 days, followed by immersion in 50% glycerol for imaging. The heads were stored in 100% glycerol post imaging. Phenotyping was conducted by photographing the right lateral, superior and palatal views of cleared heads using a Leica DFC7000T microscope camera. Skeletal preparations from each virus type were analyzed for changes in the size or shape of bones derived from the frontonasal mass, missing bones or qualitative reduction in ossification observed as reduced Alizarin Red staining. Statistical analysis was performed using the contingency analysis χ^2^ test in GraphPad Prism 10.1.0.

### Primary cultures of frontonasal mass mesenchyme

Stage 24 chicken embryos were extracted from the eggs and their extra-embryonic tissues were removed in ice-cold PBS. The frontonasal mass was dissected in cold Hank's balanced saline solution (HBSS) (without calcium and magnesium) (Thermo Fisher Scientific, #14185052) with 10% FBS and 1% antibiotic-antimycotic (Life Technologies, #15240-062). Dissected frontonasal pieces were incubated in 2% trypsin (Gibco) at 4°C for 1 h. HBSS was added to inhibit the enzymatic activity of trypsin. Ectoderm was manually peeled off from the frontonasal mass pieces. The cell solution was then centrifuged at 1000 ***g*** and 4°C for 5 min. The supernatant was removed and the frontonasal mass pieces were resuspended in HBSS. The mesenchymal cells were counted using a hemocytometer and 2×10^7^ cells/ml were resuspended in chondrogenic medium (micromass medium) containing DMEM/F12 (Corning, #10-092-CV) supplemented with 10% FBS, 1% L-glutamine (Thermo Fisher Scientific, #25030), ascorbic acid (50 mg/ml) (Thermo Fisher Scientific, #850-3080IM), 10 mM β-glycerol phosphate (Sigma-Aldrich, #G9422) and 1% antibiotic-antimycotic (Thermo Fisher Scientific, #15240-062). The cells in suspension were subsequently infected with 3 µl of viruses containing *GFP* (control), wt h*FZD2* or h*FZD2* variants (*1301G>T* or *425C>T*). The 10 µl of cell suspension infected with the virus was plated as micromass cultures (three to four spots per 35 mm culture dish, NUNC, #150318) at a density of 2×10^7^ cells/ml, (Hosseini-Farahabadi et al., 2013; Richman and Tickle, 1989, 1992; Underhill et al., 2014). The culture plates were incubated at 37°C and 5% CO_2_ for 90 min to allow cells to attach and then flooded with 2 ml of micromass medium. After that, micromass culture medium was changed every other day for experimental time points of days 4, 6 and 8.

### Wholemount staining of micromass cultures

On days 4, 6 and 8, cultures were fixed in 4% PFA for 30 min at room temperature and subjected to wholemount staining. To detect cartilage mineralization using an alkaline phosphatase stain (Table S8), fixed cultures were incubated at room temperature in 100 mM Tris for 30 min (pH 8.3). Following this, the cultures were stained with 0.5% Alcian Blue in 95% ethanol: 0.1 M HCl (1:4) to detect the area occupied by cartilage, as previously described (Hosseini-Farahabadi et al., 2013; Underhill et al., 2014). All cultures were counterstained with 50% Shandon's Instant Hematoxylin (Thermo Fisher Scientific, #6765015). The stained cultures were photographed under standard illumination using a stereomicroscope (Leica, #M125). Wholemount staining was conducted on three biological and three technical replicates, and the experiment was repeated five times.

### Histology and immunofluorescence

Embryos collected at stage 28 (Table S5) or micromass cultures (days 4, 6 and 8, Table S7) were fixed in 4% PFA. The embryo samples were immersed in the fixative for 2-3 days at 4°C. The RCAS-infected cultures were fixed in 4% PFA for 30 mins. The cultures were removed from the plate using a cell scraper (Thermo Fisher Scientific, #08-100-241), embedded in 2% agarose (Sigma-Aldrich, #A9539) on a cold ice slab, and wax embedded. The embryos (positioned frontally) and cultures (positioned transversely) were embedded in paraffin wax and sliced into 7 µm sections using a Spencer 820 microtome (American Optical Company, PA, USA). The sections were then used for histological and immunostaining analysis.

Selected frontal (embryos) and transverse (micromass cultures) sections were stained to visualize the differentiated cartilage and bone. Sections were dewaxed in xylene, rehydrated from 100% ethanol to water, and stained with 1% Alcian Blue 8GX (in 1% acetic acid) for 30 min. After staining, sections were rinsed in 1% acetic acid and water. Subsequently, sections were stained in Picrosirius Red (0.1% Sirius Red F3B in saturated picric acid) for 1 h in the dark, followed by rinsing in 1% acetic acid, dehydration through ethanol, back to xylene, and mounted with Shandon Consul-mount (Thermo Fisher Scientific, #9990441).

Immunofluorescence analysis was conducted on *in vivo* and day 6 and 8 cultures. Specific antibodies and treatments performed for each assay are outlined in Table S9. Samples were allowed to incubate in primary antibodies overnight at 4°C, and they were incubated in secondary antibodies at room temperature for 1.5 h unless otherwise specified. Sections were counterstained with Hoechst 33258 (10 μg/ml, Sigma-Aldrich, #B1155), incubated for 30 min at room temperature, then mounted with Prolong Gold antifade (Thermo Fisher Scientific, #P36930). Fluorescence images were captured using a 20× objective on a slide scanner (3DHISTECH, Budapest, Hungary).

### Western blotting to determine the antigen size recognized by PY489 antibody

Chicken DF-1 cells were transfected with proviral RCAS constructs (*GFP* or wt*DVL1*). After a month of passaging, cells were washed with 1× PBS and cell lysates were prepared subsequently using cell lysate buffer, consisting of RIPA buffer with SDS containing mini protease inhibitor cocktail (Roche, #04693124001). Lysed cells were detached using a cell scraper (Corning, #3010), transferred to a 1.5 ml Eppendorf tube, kept on ice for 15 min and centrifuged at 14,000 ***g*** at 4°C for 15 min. Supernatants were stored at −20°C. Sample buffer was added to give a protein concentration of 1.25 mg/ml. β-mercaptoethanol was added to each sample prior to loading (13.33%, BDH, #UN2966). A total of 40 µg of protein was loaded per lane. The samples were resolved on 10% SDS acrylamide gels and semi-dry transferred to a 0.45-μm nitrocellulose membrane (Thermo Fisher Scientific, membrane #88018; 50 V for 90 min in a Trans-Blot SD semi-dry Transfer Cell, Bio-Rad, #170-3940). The membranes were blocked for 1 h in 5% skim milk in 1× PBS. The primary antibody anti-β-catenin was added to blocking buffer and incubated overnight at 4°C (Developmental Studies Hybridoma Bank, PY489, 0.5 µg/ml). LI-COR IRDye secondary antibody (goat anti-mouse IgG, #926 32210 800) was added at 1:10,000 and incubated for 1 h at room temperature in the dark. Subsequently, the membranes were scanned using a near-infrared LI-COR Odysseys DLx imager. We observed a major band at ∼86 kDa, the expected molecular mass of chicken nuclear β-catenin (Fig. S8).

### Apoptosis and cell proliferation

Apoptosis was analyzed using TUNEL assay on sections obtained from virus-infected frontonasal mass at stage 28 and micromass cultures sections at days 6 and 8. The TUNEL assay was performed using ApopTag Plus *in Situ* Apoptosis Fluorescein Detection Kit (Millipore Sigma, #S7111).

For cell proliferation studies, embryos at stages 28, 29 or 30 were labeled with 50 µl of 10 mM BrdU (Sigma-Aldrich, #B5002) and incubated at 38°C for 1 h before euthanizing. For labeling micromass cultures, 50 µl of 10 mM BrdU was added to the culture medium (at 37°C with 5% CO_2_) for 1 h, before fixing day 6 and day 8 cultures in 4% PFA. Immunostaining was performed on the sections with anti-BrdU (Developmental Studies Hybridoma Bank, 1:20, #G3G4) as described in Table S9. Fluorescence images were collected with a 20× objective on a slide scanner (3DHISTECH).

### qRT-PCR on frontonasal mass *in vivo* and *in vitro*

Viral spread in the frontonasal mass was quantified using primers specific to h*FZD2* (primer set, Table S10). Three biological replicates containing five to six pieces of the right half of the frontonasal mass pooled in each sample were harvested for each virus at stages 28 (E5.5) and 30 (E7.5). Similarly, three biological replicates containing pools of 12 micromass cultures per replicate were collected on days 6 and 8. Total RNA was isolated from frontonasal masses using an RNAeasy kit (QIAGEN, Toronto, Canada, #75144). SYBR Green-based qRT-PCR (Advanced Universal SYBR Green supermix; Bio-Rad, #1725271) was carried out using an Applied Biosystems StepOnePlus instrument. qRT-PCR cycling conditions were: 95°C for 10 min; 40 cycles of 95°C for 5 s and 60°C for 20 s. Analysis used human-specific primers for *FZD2* and avian primers (Table S10). The expression of each biological replicate was normalized to 18S RNA (Applied Biosystems, #4328839). Then, the ΔCt values were used to calculate ΔΔCt relative to the average levels of expression of the gene in *GFP*-infected cultures. The ΔΔCt method was used to calculate the relative fold-change expression between h*FZD2-* and *GFP*-infected frontonasal mass as described (Schmittgen and Livak, 2008). Statistical analysis was performed with one-way ANOVA with Tukey's post hoc test in GraphPad Prism 10.0.2. A sample size calculator was used to determine how many samples would need to be included to detect a *P*-value of 0.05 80% of the time. It was necessary to collect 13 biological replicates, which was not feasible for these studies.

### Luciferase reporter assays

Transient transfections for luciferase assays were performed in HEK293T cells (0.15×10^6^ cells/ml) (Gignac et al., 2019, 2023) or untreated stage 24 frontonasal mass mesenchymal micromass cultures (1×10^7^ cells/ml) as described previously (Geetha-Loganathan et al., 2014; Hosseini-Farahabadi et al., 2013). Cells were transfected with Lipofectamine 3000 (Invitrogen, #L3000-008; NUNC 24-well plates, #142475). HEK293T cells were transfected 24 h after plating (40-50% confluence). Micromass cultures (2×10^7^ cells/ml) were allowed to attach for 45 min after plating and transfection reagents were added to the culture spot 30 min before flooding the culture plate with micromass medium. The following plasmids were used singly or in combination (totaling to 0.2 μg for HEK293T cells and 0.6 μg for frontonasal mass cultures): control/empty vector (pcDNA 3.2/V5-DEST mammalian expression vector), and plasmids containing h*FZD2*, h*FZD2^425C>T^*, h*FZD2^1301G>T^* and m*Ror2* (Addgene, #22613). Firefly reporter plasmids – Super 8× TOPFlash (STF; 0.2 μg, Addgene #12456) and ATF2 (0.4 μg) (Ohkawara and Niehrs, 2011) – were transfected along with Renilla luciferase for normalization (0.01 μg). Recombinant human WNT3A (100 ng/ml, R&D Systems, #5036-WN-010) or WNT5A (100 ng/ml, R&D Systems, #645-WN-010) protein was added 24 h post transfection.

To measure the WNT canonical/β-catenin pathway and chondrogenic activity in micromass, the cultures were infected with 3 μl of concentrated virus at the time of plating. 24 h after plating, we performed transient transfection with either STF or SOX9 luciferase (pGL3 4x48, Dr Michael Underhill, University of British Columbia, Vancouver, BC, Canada) (Weston et al., 2002). Renilla luciferase was used as a normalization control. Assay reading was performed 48 h after transfection representing day 3 of culture. The dual-luciferase reporter assay system (Promega, #E1910) was used for all luciferase assays as described (Geetha-Loganathan et al., 2014). Luminescence activity was detected with a Victor X2 Multilabel Microplate Reader (PerkinElmer) at 1 s reading with OD1 filter. All data shown represent two to three independent experiments with three technical and three biological replicates for each transfection mixture. Statistical analysis was performed using one-way ANOVA with Tukey's post hoc test in GraphPad Prism 10.0.2. The number of biological replicates was determined by our previous studies using luciferase assays (Gignac et al., 2019, 2023).

### Image analysis and statistics

For measurement of the width of the frontonasal mass at stage 28, the distance between the nasal slits (illustrated in Fig. 2T) was measured manually with the linear measurement annotation tool in CaseViewer (version 2.4, 3DHISTECH). The data were analyzed using one-way ANOVA with Tukey's post hoc test in GraphPad Prism 10.1.0.

The thickness of day 6 and day 8 micromass cultures was measured using histological sections stained with Alcian Blue and Picrosirius Red. The linear measurement annotation tool in CaseViewer (version 2.4) was used. For each experimental condition (day 6 and day 8), measurements were performed on three cultures per virus type (biological replicates). Each biological replicate represents an average of three ribbons. The data were analyzed using one-way ANOVA with Tukey's post hoc test in GraphPad Prism 10.1.0.

For analysis of immunofluorescence staining performed on stage 28 samples (*n*=3), cells in the prenasal cartilage in all treated samples were counted in a 100×100 µm^2^ area to get the average cell density. The right frontonasal mass was divided into four regions (100×250 µm^2^) (Fig. 2Q) to count the proportion of cells expressing BrdU and TUNEL. All cell counts were performed twice with the counter plugin in ImageJ by a masked observer. The data were analyzed using one-way ANOVA with Tukey's post hoc test in GraphPad Prism 10.1.0. Similar sample sizes were used in other studies on BrdU labeling (Gignac et al., 2019, 2023).

For micromass cultures, the proportion of cells expressing TWIST1 and nuclear β-catenin or cells labeled in the TUNEL assays were counted manually by the first author and a masked observer within a 500×200 µm^2^ area. The total number of TWIST1/nuclear β-catenin- or TUNEL-positive cells was divided by the total number of Hoechst^+^ cells to obtain the proportion of labeled cells. All cell counts were performed by the first author and by a masked observer with the counter plugin in ImageJ. Statistical analysis was done with one-way ANOVA with Tukey's post hoc test in GraphPad Prism 10.1.0.

## Supplementary Material

10.1242/dmm.050584_sup1 Supplementary information
