# Supplementary material for: Craniofacial studies in chicken embryos confirm the pathogenicity of human FZD2 variants associated with Robinow syndrome
Source: Dis Model Mech. 2024 Jul 5;17(6):dmm050584. doi: 10.1242/dmm.050584 (PMC11247504; doi:10.1242/dmm.050584)
Supplement: Supplementary information [file dmm-17-050584-s1.pdf]

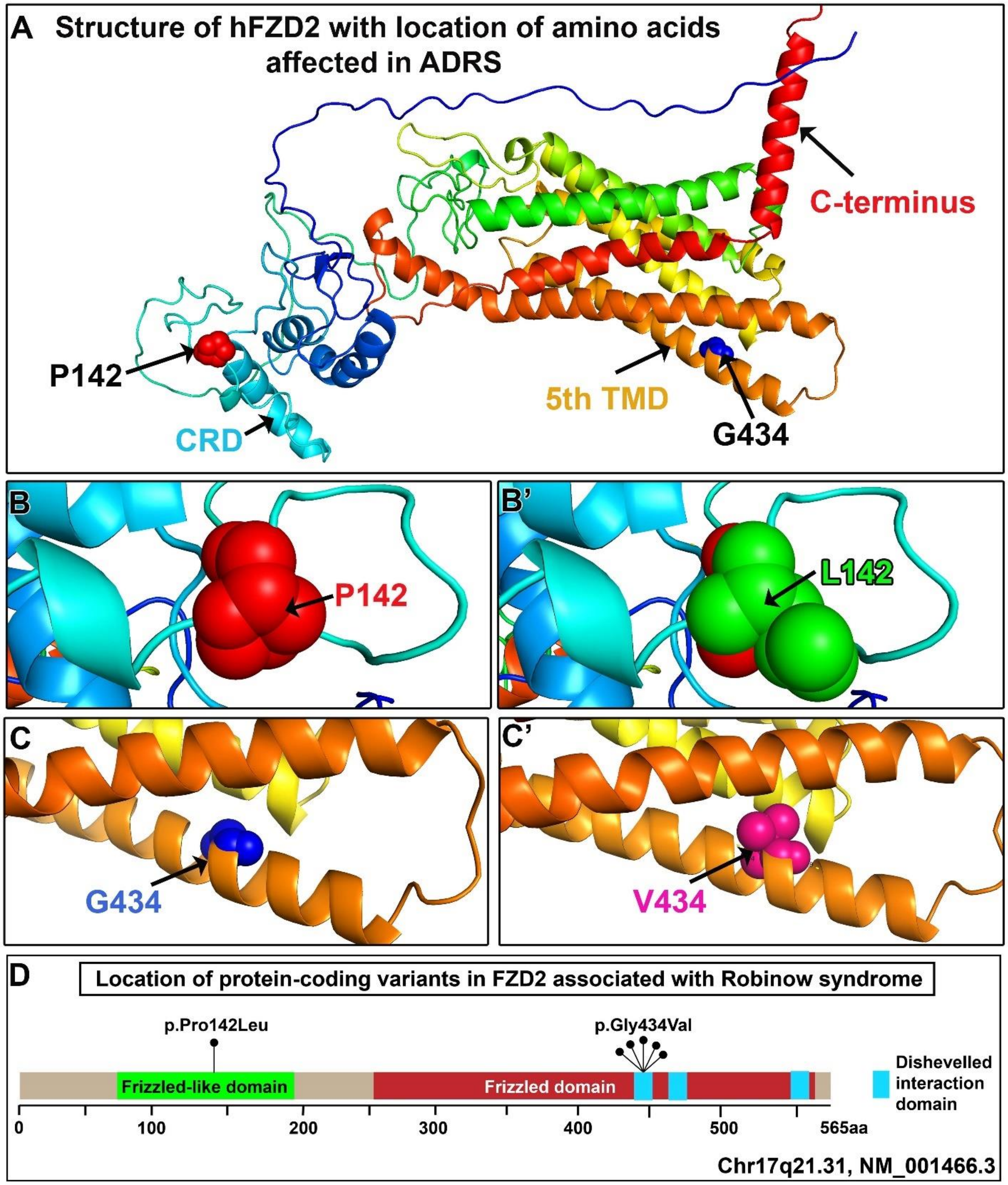

Disease Models & Mechanisms • Supplementary information

Fig. S1. Schematic representation of ADRS-associated *FZD2*$^{425C>T}$ and *FZD2*$^{1301G>T}$ variants

FZD2$^{425C>T}$ codes for P142L and *FZD2*$^{1301G>T}$ codes for G434V. (A) The predicted ribbon structure of FZD2 protein created in PyMOL software using AlphaFold (Identifier# AF-Q14332-F1). The location of affected amino acids Proline 142 (P142 red spheres) in the cysteine rich domain (cyan) and Glycine 434 (G434 royal blue spheres) in the 5th transmembrane domain (TMD) (orange) are shown. The C-terminus is represented by red. (B') Magnified view of the Proline at position 142 (red spheres) (B) replaced by Leucine (green spheres). (C') Magnified view of Glycine at position 434 (blue spheres) (C) replaced by Valine (pink spheres). (D) Representation of the known functional domains of FZD2 (green, red) and the dishevelled interaction domain (turquoise) are illustrated. The predicted location of the ADRS missense mutations p.Pro142Leu (*c.425C>T*) and p.Gly434Val (*c.1301G>T*). The number of patients classified as ADRS patients are shown with black dots (White et al., 2018; Zhang et al., 2022).

Disease Models & Mechanisms • Supplementary information

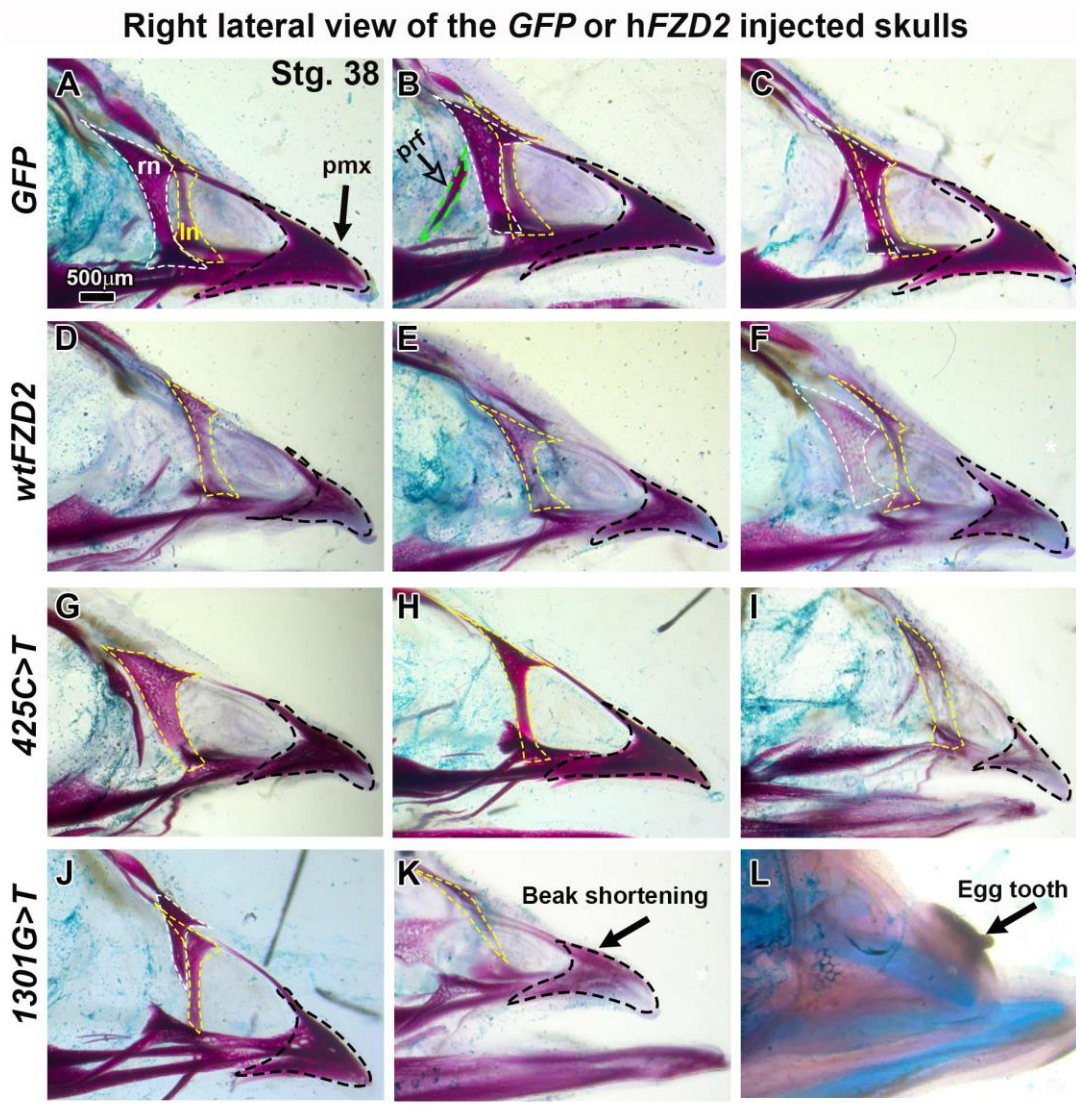

Fig. S2. Skeletal preparation of additional embryos injected with *GFP*, wild-type h*FZD2* or mutant h*FZD2* variants

Embryos injected *GFP*, wild-type h*FZD2*, h$FZD2^{425C>T}$, or h$FZD2^{1301G>T}$ into the frontonasal mass at stage 15 (E2.5) and fixed 10 days post-injection at stage 38. (A-L) Wholemount skulls cleared and stained with alcian blue (cartilage) and alizarin red (bone). (A-C) *GFP* virus allowed normal patterning and ossification of frontonasal mass derived bones including premaxillary, nasal and prefrontal. (D-L) Embryos injected with wild-type h*FZD2* or mutant h*FZD2* viruses had no defects in the upper beak morphology but had missing nasal bone on the injected side (white dashed line – right nasal bone) while the nasal bone was present on the left side (yellow dashed line). The prefrontal bone (green dashed line) was absent in >90% of embryos and the premaxillary bone (white dashed line) was present but showed faint alizarin red staining. The size of the premaxilla appeared to be smaller compared to *GFP* controls. (K, L) Some embryos injected with h$FZD2^{1301G>T}$ had beak shortening and deviation (6/19). (L) An embryo with unossified skull and short upper beak injected with h$FZD2^{1301G>T}$. The faint cartilage stain (blue) in all skulls is a technical error. Key: ln, left nasal; pmx, premaxilla; prf, prefrontal; rn, right nasal. Scale bar: A-L= 2mm.

Disease Models & Mechanisms • Supplementary information

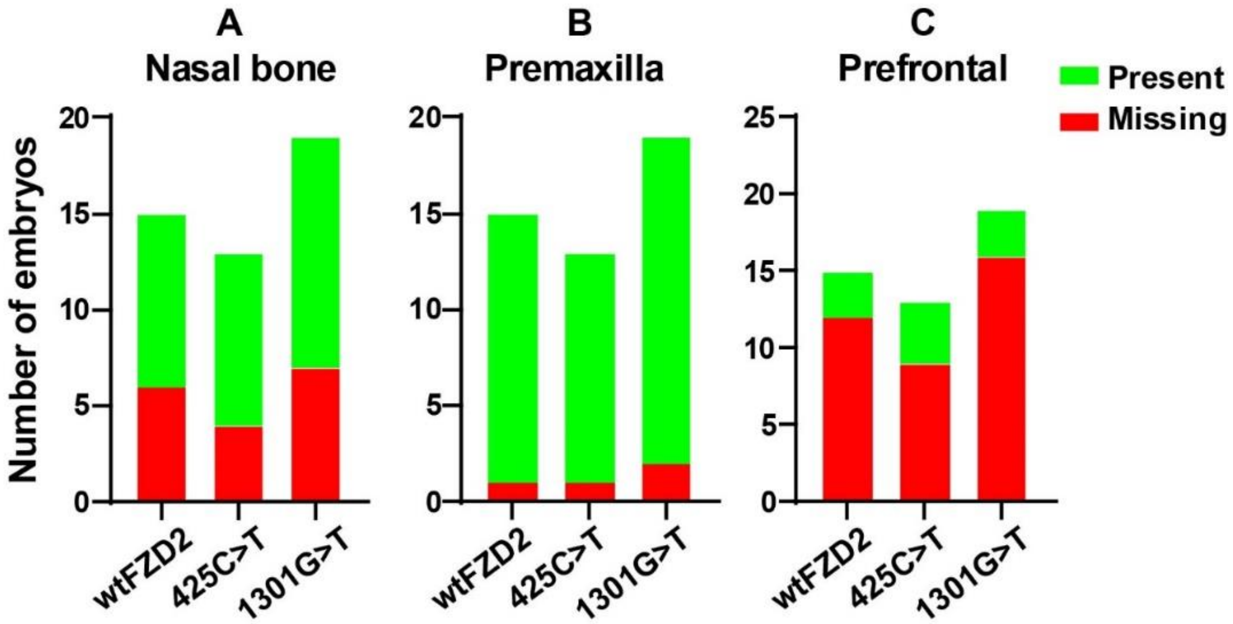

Fig. S3. Analysis of missing bones in embryos injected with human *FZD2* viruses Contingency analysis (Fisher's exact test) showing total embryos with normal or missing (A) nasal (B) Premaxillary, or (C) Prefrontal bones at stage 38 in vivo.

Disease Models & Mechanisms • Supplementary information

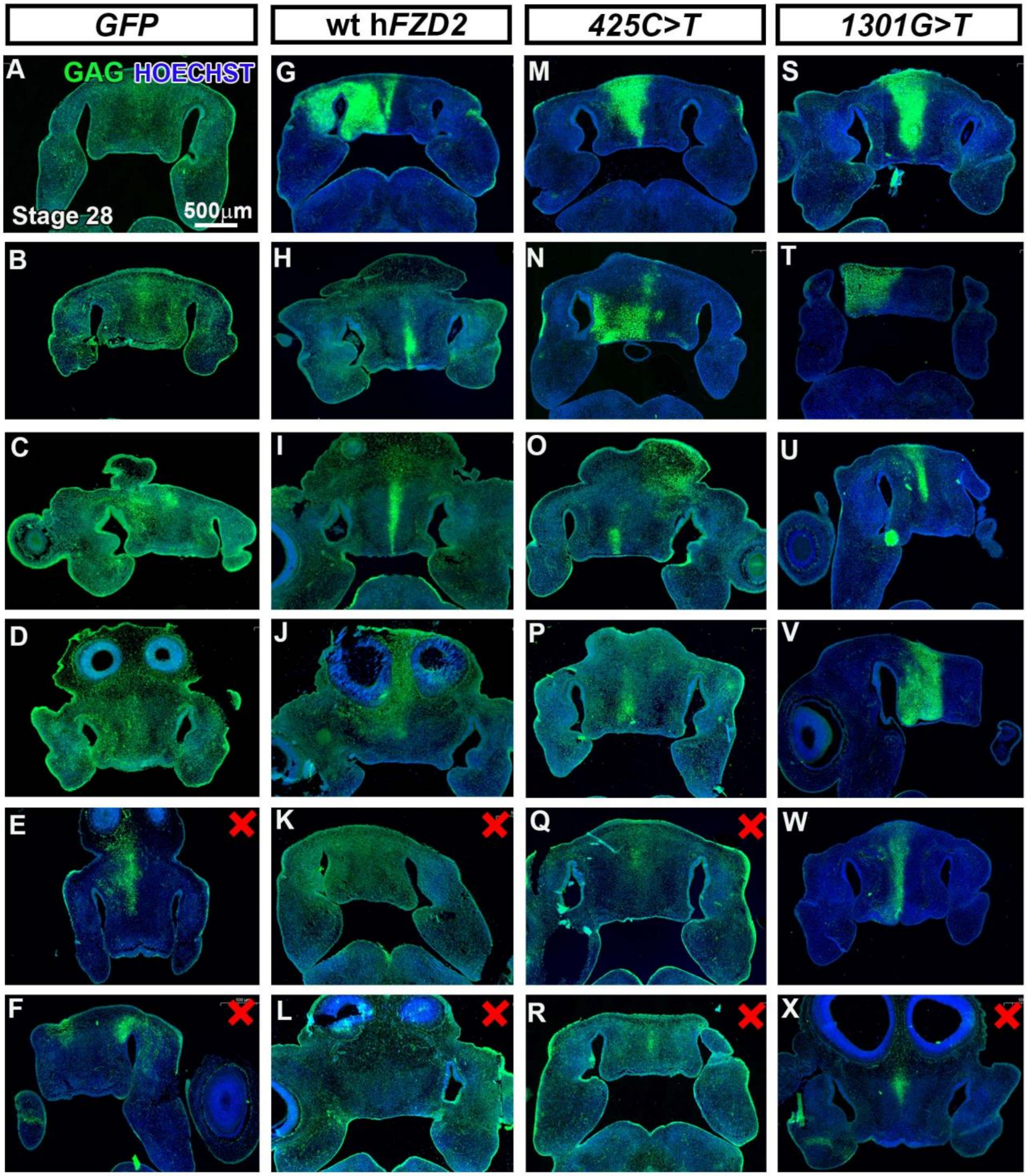

Fig. S4. Embryos at stage 28 (E5.5) injected with *GFP*, wt h*FZD2* or h*FZD2* variants

Paraffin-embedded serial sections of stage 28 embryos stained with anti-GAG antibody (green fluorescence) and counter-stained with Hoechst (blue) to detect the viral spread in the frontonasal mass. Embryos injected with (A-F) *GFP* (G-L) wt h*FZD2*, (M-R) *425C>T*, (S-X) *1301G>T* viruses Note: Sections shown in S4A, G, M, S are exactly the same sections as in Fig. 2A-D respectively. We reproduced the images so that all specimens in the study are represented. Please refer to Table S12 for a detailed list of embryos analyzed at stage 28. We also show examples of embryos with lower GAG-staining (red cross) that were not used for molecular analysis. Scale bar = 500µm

Disease Models & Mechanisms • Supplementary information

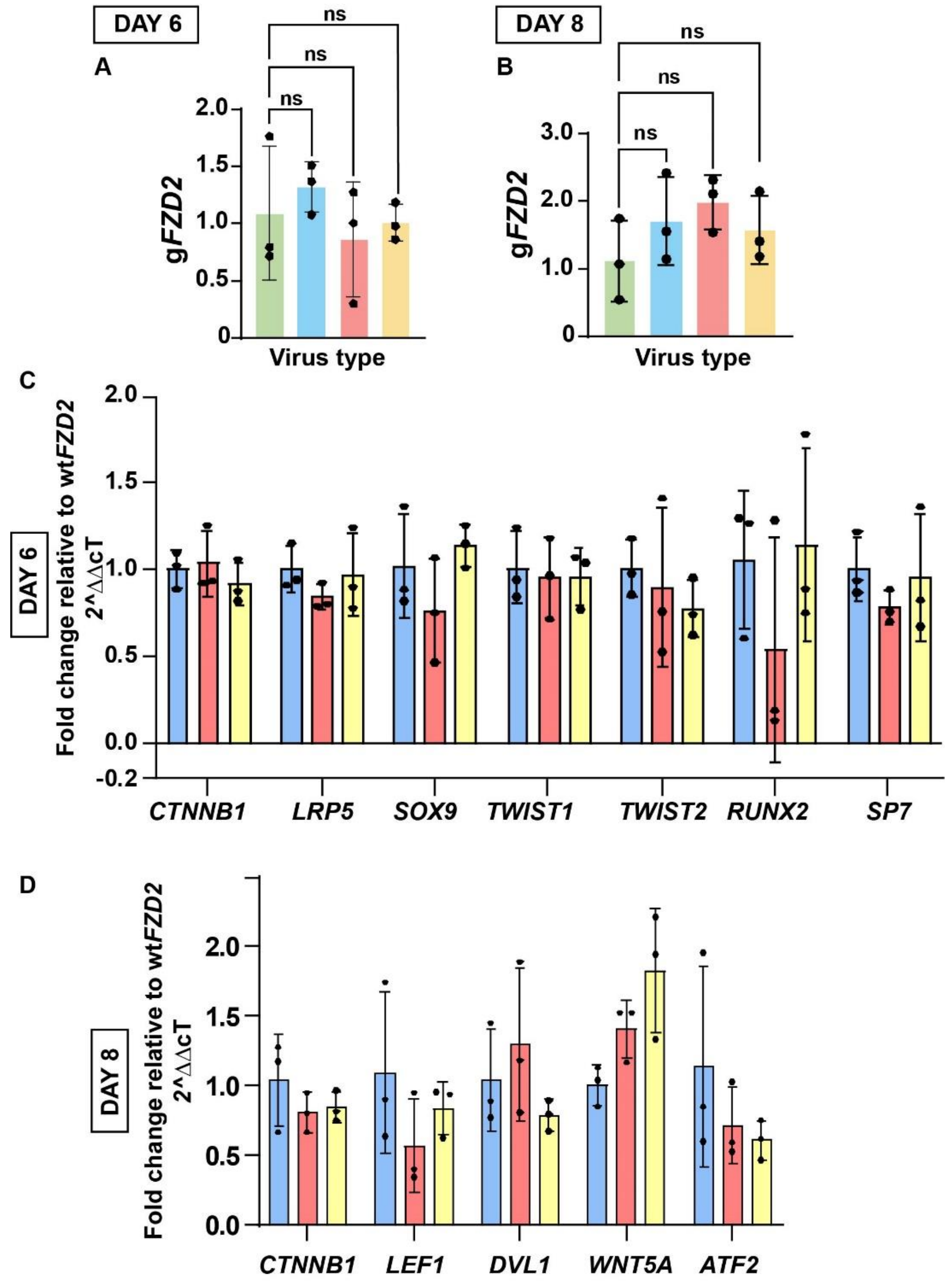

Fig. S5. qRT-PCR analysis showing effects of h*FZD2* viruses on mediators of the WNT pathway and skeletogenic mediators

RNA-isolated from day 6 or day 8 micromass cultures, three biological replicates per virus type containing pool of 8-9 micromass cultures per biological replicate. The expression of each biological replicate was normalized to 18s RNA and then these ΔCt values were used to calculate the ΔΔCt relative to the average levels of expression of the gene in *GFP*. The graphs showing gene expression changes on (A) day 6 and (B) day 8. All statistical analysis was done using one-way ANOVA, Dunnett's multiple comparison test in GraphPad Prism 10.1.0. The error bars represent one standard deviation. Genes showing statistically significant differences are shown in red text. Black dashed line represents control *GFP*. Key: Each black dot represents a biological replicate. The p values are displayed with red asterisk on the graph. * - $p < 0.05$.

Disease Models & Mechanisms • Supplementary information

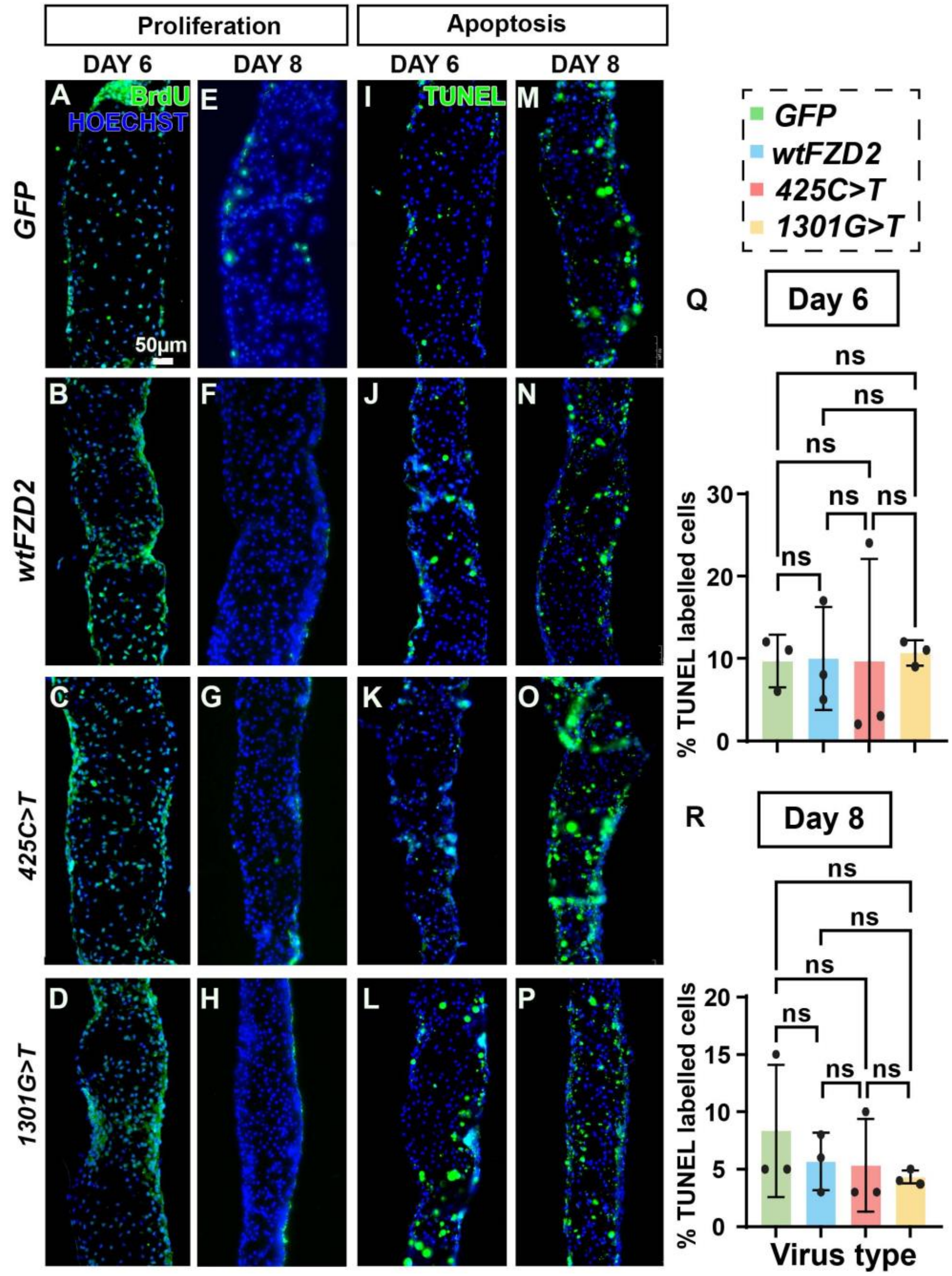

Fig. S6. qRT-PCR analysis showing effects of h*FZD2* viruses on mediators of the WNT pathway and skeletogenic mediators

RNA-isolated from day 6 or day 8 micromass cultures, three biological replicates per virus type containing pool of 8-9 micromass cultures per biological replicate. The expression of each biological replicate was normalized to 18s RNA and then these ΔCt values were used to calculate the ΔΔCt relative to the average levels of expression of the gene in *GFP*. The graphs showing gene expression changes on (A) day 6 and (B) day 8. All statistical analysis was done using one-way ANOVA, Dunnett's multiple comparison test in GraphPad Prism 10.1.0. The error bars represent one standard deviation. Genes showing statistically significant differences are shown in red text. Black dashed line represents control *GFP*. Key: Each black dot represents a biological replicate. The p values are displayed with red asterisk on the graph. * - $p < 0.05$.

Disease Models & Mechanisms • Supplementary information

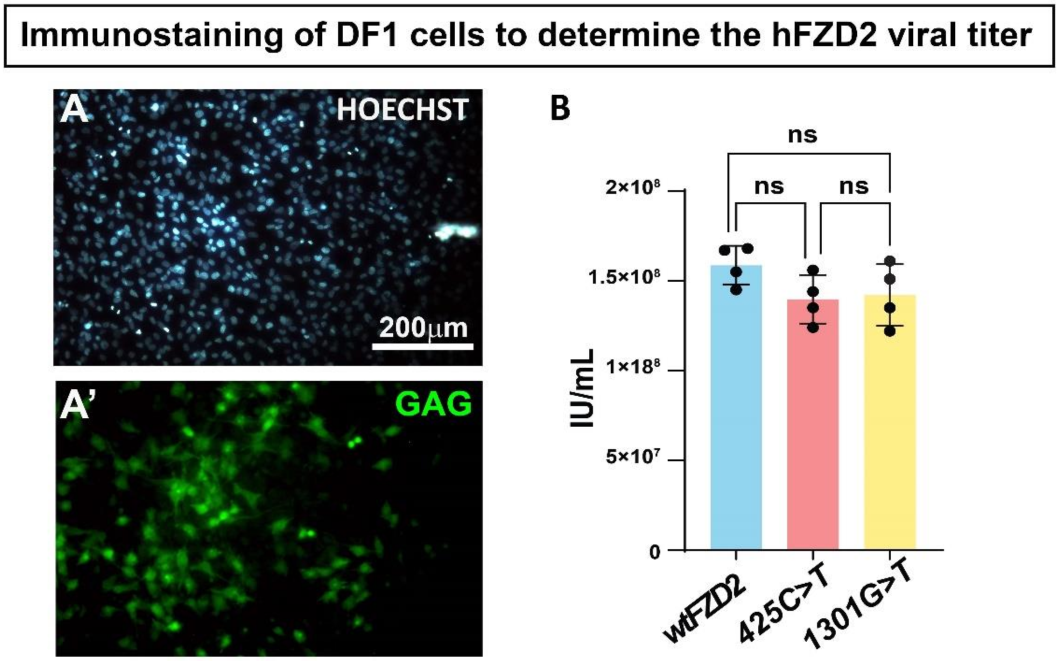

Fig. S7. Immunostaining on DF1 cells infected with high titer h*FZD2* virus and quantification of viral titer

(A, A') Immunocytochemistry performed on DF1 chicken fibroblasts infected with 2µl of high titre viral stock were fixed at 36h post-infection. The cells were stained with anti-GAG antibody (virus) and counterstained with Hoechst. (B) Quantification of viral titer showed that all h*FZD2* viruses had higher than the recommended titer of 1 x 10^7IU/mL for performing overexpression studies. The error bars represent one standard deviation. Scale bar = 200µm. Key: IU – Infectious units, ns – not significant

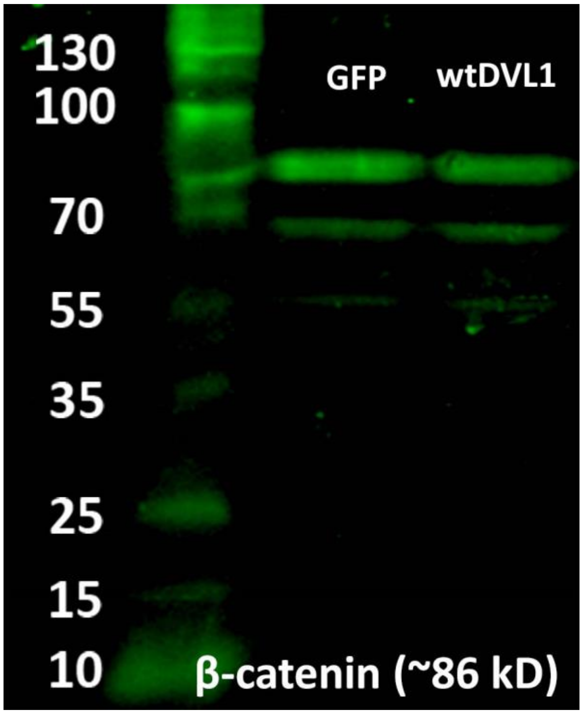

Fig. S8. The specificity of the DSHB nuclear β-catenin was tested in western blots loaded with chicken cell lysate. DF1 chicken fibroblasts transfected with proviral plasmids containing either GFP or wild-type human DVL1. Cells were cultured for 4 weeks before lysis. Blot probed with anti-nuclear β-catenin (DSHB, PY489) at 0.5 μg/ml. There is a major band at ~86 kDa coinciding with the expected molecular weight of chicken β-catenin from UniProt. The lower bands are likely non-specific.

Disease Models & Mechanisms • Supplementary information

Table S1. List of WNT pathway genes associated with Robinow Syndrome (White et al., 2015, 2016, 2018, Zhang et al. 2022)

Gene name	Inheritance pattern	Function	Variant type	OMIM
WNT5A	Autosomal dominant	Ligand	Missense	#180700
DVL1	Autosomal dominant	Adaptor protein	-1 frameshift	#616331
DVL2	Autosomal dominant	Adaptor protein	+1 frameshift	No entry
DVL3	Autosomal dominant	Adaptor protein	-1 frameshift	#616894
FZD2	Autosomal dominant	Receptor	Missense, nonsense	No entry
ROR2	Autosomal recessive	Receptor	Missense, nonsense	#268310
NXN	Autosomal recessive	Stabilizer of *DVL*	Deletion, missense	No entry

Table S2. ADRS associated missense and truncating *FZD2* variants reported in Zhang et al., 2022

	Total # of individuals	Phenotypes
**Total ADRS-FZD2 individuals from (Zhang et al., 2022)**	17	
**Missense mutations**	7	Broad forehead 7/7<br>Midface hypoplasia 7/7<br>Hypertelorism 7/7
**Truncating mutations**	9	Broad forehead 6/7<br>Midface hypoplasia 5/7<br>Hypertelorism 2/7

Table S3. Analysis of upper beak skeleton at stage 38 (E12.5) skulls stained in wholemount

RCAS Virus type injected at stage 15 (E2.5)	Stage 38 (E12.5)	External beak defects (N)	Internal bone phenotypes (N)	Missing bones			Reduced bone stain		
				pmx	n	prf	pmx	n	prf
GFP	21	0	0	0	0	0	0	0	0
**wild-type h*FZD2***	15	1	12	1	6	12	9	4	-
425C>T	13	1	9	1	4	9	1	3	-
1301G>T	19	6	16	2	7	16	5	4	-

1 – Total embryos analyzed for skeletal phenotypes caused by the FZD2 viruses
2- Total number of embryos with external phenotypes including short or deviated upper beak observed externally
3 – The total number of samples with a phenotype was analyzed in detail and classified as embryos with missing bones or faint or absent alizarin red staining in bones derived from the frontonasal mass
Key: n; nasal bone, N; total sample size, pmx; premaxillary bone, prf; prefrontal bone, - missing bone, therefore ossification changes are not quantified

Table S4. Survival of in vivo injected embryos

Virus type	Total injected embryos	Total embryos survived to day 10	% survival
GFP	42	31	73%
**wild-type h*FZD2***	36	23	64%
425C>T	41	25	61%
1301G>T	42	26	62%

Disease Models & Mechanisms • Supplementary information

Table S5. Percentage of BrdU and TUNEL positive cells in the frontonasal mass at stage 28 (3 days post-injection)

Virus type	Total injected	Total survived to day 3 (stg. 28)	Total embryos expressing GAG in frontonasal mass	% BrdU positive cells in the prenasal cartilage area (stg. 28)	% TUNEL positive cells in the prenasal cartilage area (stg. 28)
GFP	8	8	7	6%	2%
**wild-type h*FZD2***	9	9	6	7%	3%
425C>T	12	12	6	5%	2%
1301G>T	11	9	8	9%	2%

Table S6. Restriction-free Primer sequence for cloning *FZD2* variants into human wt*FZD2*

***FZD2* constructs**	**Primer sequence (5'→ 3')**
425 C→T	Fw: GAGCACTTCCTGCGCCACG<br>Rev: CGCGGCCCGATGGTTCC
1301 G→T	Fw: CCTCCTGGCCGTCTTCGTGTCGCTC<br>Rev: GCACTACACGCCGCGCATGTCG

Table S7. Micromass cultures used for histology and immunostaining analysis

Virus type	Total cultures collected for histology/immunostaining		
	**Day 4**	**Day 6**	**Day 8**
GFP	3	7	6
**wild-type h*FZD2***	3	7	4
425C>T	3	9	4
1301G>T	3	7	6

Table S8. Alkaline Phosphatase stain for staining mineralized fibroblasts in micromass

**Ingredient name**	**Distributor name, Product #**	
**Naphthol AS-MX phosphate**	Sigma Aldrich; N48751	1mg/ml dissolved in dimethyl sulfoxide
**Fast red violet LB salt**	Sigma-Aldrich; F3381	6mg/ml
**1M Tris (pH 8.3)**	Sigma; 77-86-1	10%

Table S9. Antibodies and immunofluorescence reagents

Antigen Retrieval (steam 15 minutes, 95°C)	Permeabilization/ Pre-treatment	Blocking serum	Primary antibody, source, dilution (Overnight, 4°C)	Secondary Antibody and counterstain
10mM Sodium Citrate	0.1% Triton X-100 in 1XPBS for 15 min	10% Goat serum (Sigma G9023), 0.1% tween-20 in 1X PBS, (90 minutes, RT)	**Ctnnb1/PY489-ß-catenin** Developmental Studies Hybridoma bank (DSHB), 5µg/ml, Mouse monoclonal	ThermoFisher, Cy5 goat anti-mouse, #A10524,<br><br>ThermoFisher, Cy5 goat anti-rabbit, #A10523,<br><br>ThermoFisher, Alexa Fluor™ 488 goat anti-mouse, A11029,<br><br>ThermoFisher, Alexa Fluor™ 488 goat anti-rabbit A11034, 1:200 at RT in dark, 90mins<br><br>**Hoechst** 10µg/ml (Sigma 33568) in 1 X PBS (RT in dark for 30 min)<br><br>*Coverslip with ProLong™ Gold Antifade mountant (Invitrogen)
10 mM sodium citrate	0.5% hyaluronidase in Hank's Balanced Salt Solution, 45min (following antigen retrieval)		**Collagen type II** DSHB, #II-II6B3, 5µg/ml, Mouse monoclonal	
10mM Sodium Citrate	0.5% hyaluronidase in Hank's Balanced Salt Solution, 45min (following antigen retrieval)		**Collagen type II** Proteintech®, 28459-1-AP, 1:50, Rabbit polyclonal	
1X Diva Decloaker (BioCare Medical)	0.1% Triton X-100 in 1XPBS for 15 min		**Gag-pro AMV-3C2** DSHB, 1:4, Mouse monoclonal	
10mM Sodium Citrate/ 1X Diva Decloaker (BioCare Medical)	0.1% Triton X-100 in 1XPBS for 15 min		**SOX9** Sigma-Aldrich, #HPA001758 1:200, Rabbit Polyclonal	
10mM Sodium Citrate/ 1X Diva Decloaker (BioCare Medical)	0.1% Triton X-100 in 1XPBS for 15 min		**TWIST1** Twist2C1a Abcam #ab50887 1:50, Mouse monoclonal	
0.3% Triton X-100 in 1XPBS for 15 min	0.1% Triton X-100 in 1XPBS for 15 min		**Flag (DYKDDDDK)** Thermo Fisher PA1-984B, 1µg/ml, Rabbit Polyclonal	
1X Diva Decloaker (BioCare Medical)	0.1% Triton X-100 in 1XPBS for 15 min		**BrdU** DSHB, G3G4, 5µg/ml, Mouse monoclonal	

Disease Models & Mechanisms • Supplementary information

Table S10. qRT-PCR primers

Gene	Accession #	Forward Primer	Reverse Primer
hFZD2	NM001466	CTTCCTGTGCTCCATGTACG	GCCACTGAAAACCGAACTTG
gFZD2	NM204222.2	CTCCCATTGAGAGGACAGGT	TGTTTCGTTGTCCAGTCCAT
gDVL1	XM 015297120.1	CTCCCATTGAGAGGACAGGT	TGTTTCGTTGTCCAGTCCAT
gWNT5A	NM003392	CAATGGCTTCTCAGTACCTCG	ACATCTGCACAGGGTTCATG
gCTNNB1	NM 205081	CTTGGACTTGACATTGGTGC	CAGAGTGGAAAGAACGGTAGC
gROR2	NM 001080716.1	AGTGCTGGAATGAATTTCCC	GGGAACTTGTTTGTGTGGTG
gLRP5	NM001012897	ACCAAAGCCAGAACCCAG	CAGCACCATCCCTATTGACTC
gATF2	NM 204904	GCCAGCGTTTTACCAATGAG	AGTTGGTGTTGGTGTCTGATC
gLEF1	NM_001398055.1	CATCAAGTCCTCGCTGGTC	GCCCTTGTCATGGTAGGAATC
gCOL2A1	NM 204426.1	GGACCAGCAAGACGAAAGAC	CGTAGCTGAAGTGGAAACCG
gSOX9	NM_204281.2	CTGGGCAAGCTGTGGAG	GGTTGGTACTTGTAGTCGGG
gTWIST1	NM_204739	GACTCCAAGATGGCAAGCTG	CTCCATTCTCCACACCGAGA
gTWIST2	NM_204679	GAGTTATGCCTTCTCAGTCTGG	ACGTCCCAATTCCACTTCAG
gBMP2	NM204358	GCTGTTTTGAGGTGGATTGC	AGGCACTGTTCTCTTTGTCC
gBMP7	XM 417496	GTCAAACATCGCAGAGAACAG	TCCTTCACAGTAATACGCAGC
gNOGGIN	NM204123	ACTTTATGGCTATGTCCCTGC	AACTCCAGCCCCTTGATTTC
gBMPER	NM 001007080	AGCTGTCCTCATGGTAAAATCC	AACGTACTGACATGTTCCCTG
gSP7	XM015300329.3	GTTCGTCTGCAATTGGCTCT	AATTTCTTCTCGCGGGTGTG
gRUNX2	NM204128	ACCTAGTTTGTTCCCTGAACG	GTAATCTGACTCTGTCCTTGTGG
gMSX1	NM_204559	TTCGGTCAAATCGGAGAACTC	TTCGTCTTGTGCTTCCTCAG

Table S11. List of Stage 28 embryos used in Figure 2 and Figure S4

Figure number	Antibody	Embryo number	Ribbon, section number	Channel	Virus type
2A	GAG	ST87	R17, S9	Alexa fluor 488	*GFP*
2B	GAG	ST122	R10, S9	Alexa fluor 488	*WT FZD2*
2C	GAG	ST115	R7, S9	Alexa fluor 488	*425C>T*
2D	GAG	ST104	R8, S9	Alexa fluor 488	*1301G>T*
2A’	SOX9	ST87	R17, S9	Cy5	*GFP*
2B’	SOX9	ST122	R10, S9	Cy5	*WT FZD2*
2C’	SOX9	ST115	R7, S9	Cy5	*425C>T*
2D’	SOX9	ST104	R8, S9	Cy5	*1301G>T*
2A’’	GAG+SOX9	ST87	ST87	Alexa fluor 488, Cy5	*GFP*
2B’’	GAG+SOX9	ST122	ST122	Alexa fluor 488, Cy5	*WT FZD2*
2C’’	GAG+SOX9	ST115	ST115	Alexa fluor 488, Cy5	*425C>T*
2D’’	GAG+SOX9	ST104	ST104	Alexa fluor 488, Cy5	*1301G>T*
2E	TWIST1	ST88	R7, S9	Alexa fluor 488	*GFP*
2F	TWIST1	ST122	R10, S8	Alexa fluor 488	*WT FZD2*
2G	TWIST1	ST115	R7, S8	Alexa fluor 488	*425C>T*
2H	TWIST1	ST94	R9, S9	Alexa fluor 488	*1301G>T*
2I	BrdU	ST88	R7, S8	Alexa fluor 488	*GFP*
2J	BrdU	ST122	R10, S7	Alexa fluor 488	*WT FZD2*
2K	BrdU	ST115	R7, S7	Alexa fluor 488	*425C>T*
2L	BrdU	ST94	R9, S6	Alexa fluor 488	*1301G>T*
2I’	BrdU	ST88	High mag. of 2I	Alexa fluor 488	*GFP*
2J’	BrdU	ST122	High mag. of 2J	Alexa fluor 488	*WT FZD2*
2K’	BrdU	ST115	High mag. of 2K	Alexa fluor 488	*425C>T*
2L’	BrdU	ST94	High mag. of 2L	Alexa fluor 488	*1301G>T*
2M	TUNEL	ST88	R5, S9	Alexa fluor 488	*GFP*
2N	TUNEL	ST122	R11, S9	Alexa fluor 488	*WT FZD2*
2O	TUNEL	ST115	R5, S9	Alexa fluor 488	*425C>T*
2P	TUNEL	ST98	R4, S9	Alexa fluor 488	*1301G>T*
2M’	TUNEL	ST88	High mag. of 2M	Alexa fluor 488	*GFP*
2N’	TUNEL	ST122	High mag. of 2N	Alexa fluor 488	*WT FZD2*
2O’	TUNEL	ST115	High mag. of 2O	Alexa fluor 488	*425C>T*
2P’	TUNEL	ST98	High mag. of 2P	Alexa fluor 488	*1301G>T*
S4A	GAG	ST87	R17, S9 (repeated in 2A - A’’)	Alexa fluor 488	*GFP*
S4B	GAG	ST88	R6, S9	Alexa fluor 488	*GFP*
S4C	GAG	ST90	R3, S9	Alexa fluor 488	*GFP*
S4D	GAG	ST91	R12, S9	Alexa fluor 488	*GFP*
S4E	GAG	ST86	R4, S9	Alexa fluor 488	*GFP*
S4F	GAG	ST92	R5, S9	Alexa fluor 488	*GFP*
S4G	GAG	ST122	R10, S9 (repeated in 2B - B’’)	Alexa fluor 488	*WT FZD2*
S4H	GAG	ST118	R4, S9	Alexa fluor 488	*WT FZD2*
S4I	GAG	ST123	R11, S9	Alexa fluor 488	*WT FZD2*

S4J	GAG	ST128	R5, S9	Alexa fluor 488	*WT FZD2*
S4K	GAG	ST124	R9, S9	Alexa fluor 488	*WT FZD2*
S4L	GAG	ST125	R7, S9	Alexa fluor 488	*WT FZD2*
S4M	GAG	ST115	R7, S9 (repeated in 2C -C’’)	Alexa fluor 488	*425C>T*
S4N	GAG	ST106	R6, S9	Alexa fluor 488	*425C>T*
S4O	GAG	ST105	R14, S9	Alexa fluor 488	*425C>T*
S4P	GAG	ST108	R5, S9	Alexa fluor 488	*425C>T*
S4Q	GAG	ST111	R9, S9	Alexa fluor 488	*425C>T*
S4R	GAG	ST116	R6, S9	Alexa fluor 488	*425C>T*
S4S	GAG	ST104	R8, S9 (repeated in 2D - D’’)	Alexa fluor 488	*1301G>T*
S4T	GAG	ST94	R9, S8	Alexa fluor 488	*1301G>T*
S4U	GAG	ST100	R7, S9	Alexa fluor 488	*1301G>T*
S4V	GAG	ST95	R11, S9	Alexa fluor 488	*1301G>T*
S4W	GAG	ST98	R5, S9	Alexa fluor 488	*1301G>T*
S4X	GAG	ST99	R5, S9	Alexa fluor 488	*1301G>T*

Table S12. List of micromass cultures used in Figure 4 and Figure 5

Figure number	Stain/Antibody	Channel	Day of culture	Culture number	Ribbon number	Virus type
4A	Alcian blue, Picrosirius red	-	6	ST1.10	R32, S10	*GFP*
4B	Alcian blue, Picrosirius red	-	6	ST2.10	R27, S10	*WT FZD2*
4C	Alcian blue, Picrosirius red	-	6	ST4.8	R37, S10	*425C>T*
4D	Alcian blue, Picrosirius red	-	6	ST3.7	R35, S10	*1301G>T*
4E	GAG	Alexa fluor 488	6	ST1.10	R21, S9	*GFP*
4F	GAG	Alexa fluor 488	6	ST2.10	R26, S9	*WT FZD2*
4G	GAG	Alexa fluor 488	6	ST4.8	R37, S9	*425C>T*
4H	GAG	Alexa fluor 488	6	ST3.7	R35, S9	*1301G>T*
4I	COL2A1	Cy5	6	ST1.10	R20, S9	*GFP*
4J	COL2A1	Cy5	6	ST2.10	R27, S9	*WT FZD2*
4K	COL2A1	Cy5	6	ST4.8	R33, S9	*425C>T*
4L	COL2A1	Cy5	6	ST3.7	R35, S8	*1301G>T*
4M	Alcian blue, Picrosirius red	-	8	ST5.3	R51, S10	*GFP*
4N	Alcian blue, Picrosirius red	-	8	ST6.1	R32, S10	*WT FZD2*
4O	Alcian blue, Picrosirius red	-	8	ST8.3	R54, S10	*425C>T*
4P	Alcian blue, Picrosirius red	-	8	ST7.4	R33, S10	*1301G>T*
4Q	GAG/FLAG	Alexa fluor 488/Cy5	8	ST5.3	R51, S9	*GFP*
4R	GAG/FLAG	Alexa fluor 488/Cy5	8	ST6.1	R32, S9	*WT FZD2*
4S	GAG/FLAG	Alexa fluor 488/Cy5	8	ST8.3	R54, S9	*425C>T*
4T	GAG/FLAG	Alexa fluor 488/Cy5	8	ST7.4	R33, S9	*1301G>T*
4U	COL2A1	Cy5	8	ST5.3	R50, S9	*GFP*
4V	COL2A1	Cy5	8	ST6.1	R32, S8	*WT FZD2*
4W	COL2A1	Cy5	8	ST8.3	R52, S9	*425C>T*
4X	COL2A1	Cy5	8	ST7.4	R36, S9	*1301G>T*
5A	Nuclear β-catenin	Cy5	8	ST5.2	R53, S9	*GFP*
5B	Nuclear β-catenin	Cy5	8	ST6.4	R29, S9	*WT FZD2*
5C	Nuclear β-catenin	Cy5	8	ST8.4	R41, S9	*425C>T*
5D	Nuclear β-catenin	Cy5	8	ST7.3	R33, S9	*1301G>T*
5E	TWIST1	Cy5	8	ST5.2	R57, S9	*GFP*
5F	TWIST1	Cy5	8	ST6.4	R28, S9	*WT FZD2*
5G	TWIST1	Cy5	8	ST8.4	R42, S9	*425C>T*
5H	TWIST1	Cy5	8	ST7.3	R35, S9	*1301G>T*
5E’	COL2A1	Alexa fluor 488	8	ST5.2	R53, S8	*GFP*
5F’	COL2A1	Alexa fluor 488	8	ST6.4	R28, S8	*WT FZD2*
5G’	COL2A1	Alexa fluor 488	8	ST8.4	R42, S8	*425C>T*
5H’	COL2A1	Alexa fluor 488	8	ST7.3	R35, S8	*1301G>T*

Disease Models & Mechanisms • Supplementary information

References

White, J. J., Mazzeu, J. F., Coban-Akdemir, Z., Bayram, Y., Bahrambeigi, V., Hoischen, A., . . . Mendelian, B.-H. C., 2018. WNT Signaling Perturbations Underlie the Genetic Heterogeneity of Robinow Syndrome. American Journal of Human Genetics. 102**,** 27-43.

Zhang, C., Jolly, A., Shayota, B. J., Mazzeu, J. F., Du, H., Dawood, M., . . . Carvalho, C. M. B., 2022. Novel pathogenic variants and quantitative phenotypic analyses of Robinow syndrome: WNT signaling perturbation and phenotypic variability. HGG Adv. 3**,** 100074.
